# An observational post-authorization study to assess the effectiveness of a single dose Ad26.COV2.S for the prevention of COVID-19 using real-world data

**DOI:** 10.3389/fpubh.2024.1501919

**Published:** 2024-12-04

**Authors:** Amelia Boehme, Raymond A. Harvey, Ann Madsen, Lexie Rubens, Astra Toyip, Michael Batech, Deborah Ricci, Mawuli Nyaku

**Affiliations:** ^1^Aetion Inc., New York, NY, United States; ^2^Johnson & Johnson Innovative Medicine, Spring House, PA, United States

**Keywords:** COVID-19, vaccination, vaccine effectiveness, real-world evidence, Ad26.COV2.S

## Abstract

**Background:**

The goal of this FDA-committed, post authorization study was to assess the real-world effectiveness of Ad26.COV2.S in preventing observed COVID-19 disease in individuals in the United States interacting with the healthcare system who were vaccinated according to the national immunization recommendations.

**Methods:**

The study cohort consisted of individuals ≥18 years in the U.S. between March 1, 2021 and July 31, 2022. Two exposure groups were considered: those who received a single dose of COVID-19 Ad26.COV2.S vaccine and individuals who were unvaccinated. Individuals eligible for the referent group, defined as those who were unvaccinated, were identified through exact matching on age, sex, location, and Gagne comorbidity score. Propensity-score (PS) matched Cox proportional hazards models were used to evaluate COVID-19 related outcomes.

**Results:**

A total of 478,162 vaccinated, and 1,897,759 risk set sampled (RSS) and PS-matched unvaccinated referent individuals were included. The vaccine effectiveness (VE) against any observed COVID-19 disease was 20% (95% CI, 19 to 21%). VE increased as the outcome severity increased. The VE against COVID-19 related hospitalizations was 43% (95% CI, 40 to 45%). VE was highest, 53% (95% CI, 42 to 61%), against all-cause mortality temporally associated with COVID-19. The results of subgroup analyses generally showed a similar pattern as the main analyses with VE increasing in parallel with seriousness of outcomes, albeit with lower VE in groups thought to be at higher risk of COVID-19.

**Discussion:**

This population-representative cohort study in U.S. clinical practice showed that a single dose of Ad26.COV2.S is effective for at least 12 months against several COVID-19 related outcomes. Individuals who were vaccinated with a single dose of Ad26.COV2.S were at lower risk for developing COVID-19, for being hospitalized for COVID-19, and for all-cause mortality temporally associated with COVID-19 compared to unvaccinated individuals in the U.S. during Alpha, Delta, and Omicron BA.1, BA.2, BA.212.1, and BA.5 variants circulation.

## 1 Introduction

The COVID-19 pandemic was caused by the Severe Acute Respiratory Syndrome Coronavirus-2 (SARS-CoV-2) and is the first coronavirus to cause a modern pandemic. This global public health crisis drove the scientific community to develop vaccines at an unprecedented rate (1). Government initiatives in vaccine development including funding for research and development and advanced purchasing of vaccines encouraged the development of different vaccine platforms, including easily accessible single-dose vaccines (2). The J&J Innovative Medicine, formerly Janssen, single-dose COVID-19 vaccine (Ad26.COV2.S), uses a recombinant, replication-incompetent adenovirus type 26 to express the severe acute respiratory syndrome coronavirus-2 (SARS-CoV-2) spike protein (3–5). In the double-blind, placebo-controlled, Phase 3 randomized controlled study, “ENSEMBLE” (NCT04505722) (NLM 2020), which commenced in September 2020, the single dose Ad26.COV2.S vaccine was compared to a placebo to evaluate the efficacy for preventing moderate to severe COVID-19. Overall efficacy for preventing moderate to severe COVID-19 at least 14 days post-vaccination was found to be 56% [95% CI 51.3 to 60.8%] globally (70% [95% CI 60.7 to 76.9%] in the US). Higher efficacy was observed against severe disease (75% VE [95% CI 64.7 to 84.1%]) against severe-critical COVID-19 and 83% VE [95% CI 40.5 to 96.8%] against COVID-19-related death (5). In addition to the phase 3 randomized controlled study, observational studies evaluating effectiveness of the single dose Ad26.COV2.S vaccine were initiated to estimate the short- and long-term real-world effectiveness in the prevention of COVID-19. An interim study evaluating real-world effectiveness of a single administration of Ad26.COV2.S in the prevention of COVID-19 using a real-world COVID-19 enriched administrative claims database found Ad26.COV2.S was effective at preventing COVID-19 (efficacy 76% [95% CI, 75 to 77%]) and COVID-19 hospitalization for at least 6 months (efficacy 81% [95% CI, 78 to 82%]) (6).

The COVID-19 vaccine recommendations in the US have evolved since the approval of the initial study protocol. On November 19, 2021, the Centers for Disease Control (CDC) recommended the use of a booster vaccination for all adults (7). Additionally, on December 16, 2021 the CDC Advisory Committee on Immunization Practices (ACIP) conducted a benefit/risk assessment of COVID-19 vaccines and recommended that administration of Ad26.COV2.S should be restricted to certain populations, including those who were unwilling or unable (e.g., contraindicated) to receive an alternate COVID-19 vaccine (8). Despite the shift in recommendations during the period of dominance by the omicron variant, the results from the retrospective observational study presented here are important to estimate the effectiveness of a single administration of Ad26.COV2.S in the prevention of COVID-19 disease, specifically among at-risk subgroups.

The goal of this FDA-committed, post authorization study was to assess the real-world effectiveness of Ad26.COV2.S to prevent observed COVID-19 disease in US individuals interacting with the healthcare system who were vaccinated according to the national immunization recommendations. The three primary objectives in this study were: (1) to estimate the effectiveness of Ad26.COV2.S in preventing any observed COVID-19 (defined as COVID-19 International Classification of Diseases, Ninth Revision, Clinical Modification (ICD)-10 diagnosis and/or positive diagnostic SARS-CoV-2 nucleic acid amplification test (NAAT)); (2) to estimate the effectiveness of Ad26.COV2.S in preventing any COVID-19 related hospitalization; and (3) to estimate the effectiveness of Ad26.COV2.S in preventing all-cause mortality temporally associated with a COVID disease.

## 2 Methods

This was a retrospective observational, longitudinal cohort study of the real-world effectiveness of an Ad26.COV2.S vaccination for the prevention of any observed COVID-19 disease, COVID-19 related hospitalizations, and all-cause mortality temporally associated with a COVID disease.

### 2.1 Data

We analyzed de-identified patient-level claims data from the HealthVerity Marketplace, a unified database of open and closed medical and pharmacy claims, hospital transactional records for inpatient and outpatient hospital encounters, and laboratory data for individuals in the US from 1 March 2020 through 18 September 2022. The claims were anonymously linked across health data sources using a unique, encrypted, and non-identifiable patient token derived from identifiable information known only to the provider to allow for longitudinal capture of diagnosis and procedure codes across healthcare systems, COVID-19 laboratory test orders and results, and pharmacy dispensing information. This national claims database includes all major insurance types, with proportions balanced and representative of the US population.

### 2.2 Population and exposure

Individuals 18 years and older with continuous medical and pharmacy claims enrollment and at least one lab test, closed medical claim, or closed pharmacy claim in the 365 days prior to cohort entry from the HealthVerity Marketplace dataset were considered for this study. Individuals who received a single dose of Ad26.COV2.S between 1 March 2021 and 31 July 2022, entered the study cohort on the day of Ad26.COV2.S vaccination (exposed group). Individuals with documentation of receiving multiple doses of Ad26.COV2.S or booster doses of other COVID-19 vaccines were excluded. Ad26.COV2.S vaccination was identified via any medical claim, pharmacy claim, inpatient hospital encounter, or outpatient hospital encounter with the CPT code 91303 or 0031A or the NDC codes 59676-580-05, 59676-0580-05, 59676-0580-15, or 59676-580-15. Everyone who received an Ad26.COV2.S vaccine was matched on the same calendar day to as many as 10 referent individuals who had no evidence of any COVID-19 vaccination at that time. The referent group was exact matched to the vaccinated group on location (3-digit zip code), age within 4 years, sex, and general health status captured by the Gagne comorbidity score (9). To create a matched unvaccinated referent group that reflects the counterfactual experience of the vaccinated group had they not been vaccinated, parallel propensity score (PS) matching with a 1% caliper was performed after the exact matching (10). The PS model included the following variables that may be associated with severity of disease (i.e., immunocompromised status) or access to treatment (i.e., age), and as available in claims data: chronic obstructive pulmonary disease (COPD), pulmonary fibrosis, HIV infection status as defined by ICD-10-CM codes, immunocompromised status including from blood or organ transplant, liver disease, malignancies excluding non-melanoma skin cancer, moderate to severe asthma, cerebrovascular disease, chronic kidney disease, hypertension, serious heart condition, obesity, sickle cell disease, thalassemia, type 1 diabetes, type 2 diabetes, neurologic condition, number of pharmacy claims, number of medical claims, recent medical claims, recent pharmacy claims, age, sex, Gagne comorbidity score, calendar month of index date and state. Definitions provided in Supplementary Table S1.

### 2.3 Follow-up and outcomes

Follow-up for both the vaccinated and unvaccinated groups started 14 days after cohort entry and continued until the occurrence of an outcome, receipts of any COVID-19 vaccine or booster dose, death, disenrollment, or up to 18 September 2022. In addition, cases and hospitalizations within 14 days post-vaccination were censored. Medically attended COVID-19 disease was defined by either recording of an inpatient or outpatient ICD-10-CM diagnosis code of U07.1 in any position and/or a recorded positive SARS-CoV-2 diagnostic polymerase chain reaction or nucleic acid amplification test result. COVID–19–related hospitalization was defined as any inpatient medical claim or inpatient hospital encounter for which any observed COVID-19 began in the 21 days prior to the start of the hospitalization through the last day of the hospitalization. All-cause mortality during a COVID-19-related hospitalization was defined as any death observed during an inpatient hospital encounter for which any observed COVID-19 began in the 21 days prior to the start of the hospitalization through the last day of the hospitalization (Supplementary Figure S1).

### 2.4 Participant characteristics

Participant characteristics such as demographic factors and comorbid conditions were assessed in the 365-day period prior to cohort entry. The variables used in the exact matching process that were evaluated on cohort entry included age, sex, location, health insurance status. Comorbidities evaluated during the baseline period include Gagne comorbidity score, COPD, pulmonary fibrosis, HIV infection status as defined by ICD-10-CM codes, immunocompromised status including from blood or organ transplant, liver disease, malignancies excluding non-melanoma skin cancer, moderate to severe asthma, cerebrovascular disease, chronic kidney disease, hypertension, serious heart condition, obesity, sickle cell disease, thalassemia, type 1 diabetes, type 2 diabetes, neurologic condition, number of pharmacy claims, number of medical claims, recent medical claims, recent pharmacy claims, age, sex, comorbidity score, calendar month of index date and state. Definitions are provided in Supplementary Table S1.

### 2.5 Statistical analysis

After 1:10 exact matching on calendar time, location, age, sex, and comorbidity score, individuals were further matched on pre-exposure risk factors for COVID-19 severity using propensity scores (PSs) to ensure balance between the vaccinated and unvaccinated group. PSs were estimated with logistic regression including all pre-exposure patient characteristics reported in Supplementary Table S1. Each vaccinated individual was PS-matched with up to 4 unvaccinated individuals with a caliper of ±0.01. A standardized difference of <0.10 was used as criterion for evaluating balance between observed demographic and clinical characteristics between the PS-matched vaccinated and unvaccinated cohorts. Right censoring of individuals occurred at the first event of any of the following: death, loss to follow-up/disenrollment from the insurance plan, end of study period, receipt of any COVID-19 vaccine, or the end of available data from the data source.

Incidence rates per 1,000 person-years and hazard ratios (HRs) with 95% confidence intervals (CI) were calculated. The primary approach used time-to-event analyses using Cox proportional hazards models to assess endpoints, producing hazard ratios (HR) and 95% CI, for an as-treated approach. Vaccine Effectiveness (VE) in percent was computed as (1-HR) 𝗑100. Kaplan–Meier curves with 95% CIs. The proportional hazards assumption was assessed with visual inspection of Kaplan–Meier curves. Subgroup analyses were conducted in prespecified groups of interest.

## 3 Results

### 3.1 Study population

**Figure 1:**
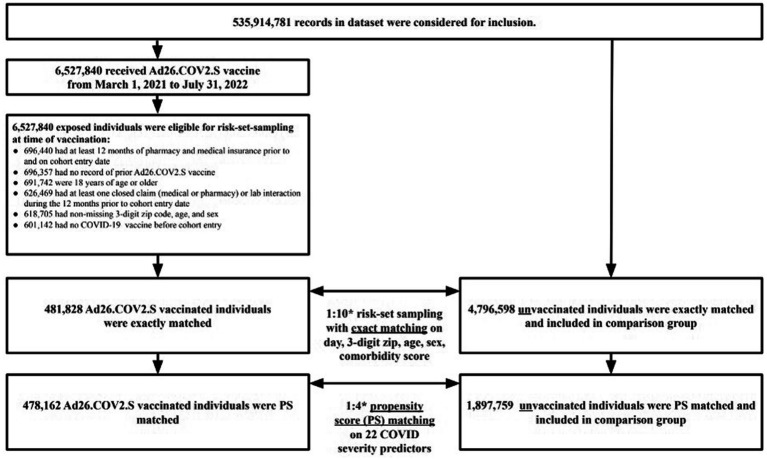
Study population and flow diagram.

The study cohort included a total of 6,527,840 patients in the dataset who received the Ad26.COV2.S vaccine between 1 March 2021 to 31 July 2022. After excluding patients who did not meet study criteria, 601,142 Ad26.COV2.S vaccinated patients were eligible for the study cohort. Of the 601,142 Ad26.COV2.S vaccinated exposed individuals, 481,828 (80%) were successfully matched to unvaccinated individuals during the 1:10 risk-set sampling. After 1:4 propensity score matching, the final study sample consisted of 478,162 Ad26.COV2.S vaccinated (exposed) individuals matched to 1,897,759 unvaccinated (referent) individuals (Figure 1).

### 3.2 Participant characteristics

Prior to PS-matching, all covariates were balanced (ASD <0.1) between the exposure and referent groups except for Commercial and Medicaid insurance (Table 1). After PS-matching, all covariates were balanced (Table 1). The mean age in our study cohort was ~44 years old in both groups. Most patients in the PS-matched cohort had Commercial insurance (58%) or Medicaid (37%). The most common comorbidities were hypertension (28.0%), obesity (21.4%), cardiovascular disease (33.1%), and immunosuppression (20.6%) (Table 1).

**Table 1:** Characteristics of individuals who received Ad26.COV2.S vaccination and matched unvaccinated individuals in the nationally representative cohort.

Characteristics	RSS matched (Pre-PS matching)^b^			RSS and PS matched^c^		
	Ad26.COV2.S vaccinated (*n* = 478,162)	Matched unvaccinated (*n* = 4,491,361)	Absolute standardized difference	Ad26.COV2.S vaccinated (*n* = 478,162)	PS-matched unvaccinated (*n* = 1,897,759)	Absolute standardized difference
Age, mean (sd)	44.57 (15.73)	44.30 (15.79)	0.018	44.57 (15.73)	44.44 (15.62)	0.008
Female sex; *n* (%)	247,395 (51.7%)	2,326,598 (51.8%)	0.001	247,395 (51.7%)	984,912 (51.9%)	0.003
Gagne combined comorbidity score, mean (sd)	0.61 (1.52)	0.60 (1.51)	0.008	0.61 (1.52)	0.57 (1.48)	0.023
Insurance type^d^						
Medicaid	176,990 (37.0%)	2,043,496 (45.5%)	0.173	176,990 (37.0%)	699,820 (36.9%)	0.003
Medicare	28,035 (5.9%)	306,617 (6.8%)	0.040	28,035 (5.9%)	111,068 (5.9%)	0.000
Commercial	278,665 (58.3%)	2,195,027 (48.9%)	0.189	278,665 (58.3%)	1,107,136 (58.3%)	0.001
Chronic pulmonary diseases; *n* (%)	52,380 (11.0%)	490,745 (10.9%)	0.001	52,380 (11.0%)	200,260 (10.6%)	0.013
Liver diseases; *n* (%)	22,430 (4.7%)	200,644 (4.5%)	0.011	22,430 (4.7%)	83,944 (4.4%)	0.013
Renal diseases; *n* (%)	14,214 (3.0%)	131,511 (2.9%)	0.003	14,214 (3.0%)	51,565 (2.7%)	0.015
Diabetes (type 1 or 2); *n* (%)	60,657 (12.7%)	557,613 (12.4%)	0.008	60,657 (12.7%)	235,675 (12.4%)	0.008
Hypertension; *n* (%)	133,864 (28.0%)	1,229,457 (27.4%)	0.014	133,864 (28.0%)	525,053 (27.7%)	0.007
Obesity; *n* (%)	102,281 (21.4%)	935,941 (20.8%)	0.014	102,281 (21.4%)	400,650 (21.1%)	0.007
Malignancy (excluding of the skin); *n* (%)	14,242 (3.0%)	129,285 (2.9%)	0.006	14,242 (3.0%)	53,369 (2.8%)	0.010
Congestive heart failure; *n* (%)	16,573 (3.5%)	158,334 (3.5%)	0.003	16,573 (3.5%)	60,224 (3.2%)	0.016
Cardiac arrhythmias; *n* (%)	37,372 (7.8%)	352,795 (7.9%)	0.001	37,372 (7.8%)	140,564 (7.4%)	0.015
Coagulopathy; *n* (%)	7,360 (1.5%)	72,237 (1.6%)	0.006	7,360 (1.5%)	26,516 (1.4%)	0.012
Peripheral vascular disorder; *n* (%)	16,932 (3.5%)	160,423 (3.6%)	0.002	16,932 (3.5%)	62,222 (3.3%)	0.014
Pulmonary circulation disorders; *n* (%)	2,457 (0.5%)	25,027 (0.6%)	0.006	2,457 (0.5%)	8,699 (0.5%)	0.008
Cerebrovascular disease; *n* (%)	6,238 (1.3%)	62,993 (1.4%)	0.008	6,238 (1.3%)	22,305 (1.2%)	0.012
Any cardiovascular disease; *n* (%)	158,118 (33.1%)	1,460,079 (32.5%)	0.012	158,118 (33.1%)	618,606 (32.6%)	0.010
Chronic neurological disorders; *n* (%)	16,356 (3.4%)	154,565 (3.4%)	0.001	16,356 (3.4%)	61,242 (3.2%)	0.011
Immunosuppression; *n* (%)	98,292 (20.6%)	847,148 (18.9%)	0.043	98,292 (20.6%)	384,211 (20.2%)	0.008

^a^Characteristics reported for population matched with risk-set sampling and propensity scores. Unless otherwise noted, demographic variables are assessed at cohort entry (index) and comorbidities and clinical utilization variables are assessed during the 1 year before cohort entry. ^b^The number of vaccinated and unvaccinated RSS Matched patients’ pre-PS matching represent patients who began follow up (i.e., were not censored between days 0–13) and therefore these numbers are different than the number of patients successfully matched to unvaccinated individual(s) during risk-set sampling. ^c^In the risk-set-sampled matching, patients were exact matched (1:10) on location (3-digit ZIP), age within 4 years, sex, and general health status captured by the Gagne comorbidity score. Propensity score modeling included matching (1:4) on variables including age, sex, Gagne Comorbidity Score, insurance status, chronic pulmonary diseases, liver disease, renal disease, diabetes (type 1 or 2), hypertension, obesity, malignancy (excluding of the skin), cardiac arrhythmias, coagulopathy, peripheral vascular disorder, pulmonary circulation disorders, cardiovascular disease, chronic neurological disorders, and immunosuppression. ^d^Patients have dual insurance status on index. These patients are reflected as a separate subgroup in our insurance status subgroup analysis.

### 3.3 Incidence and vaccine effectiveness

**Figure 2:**
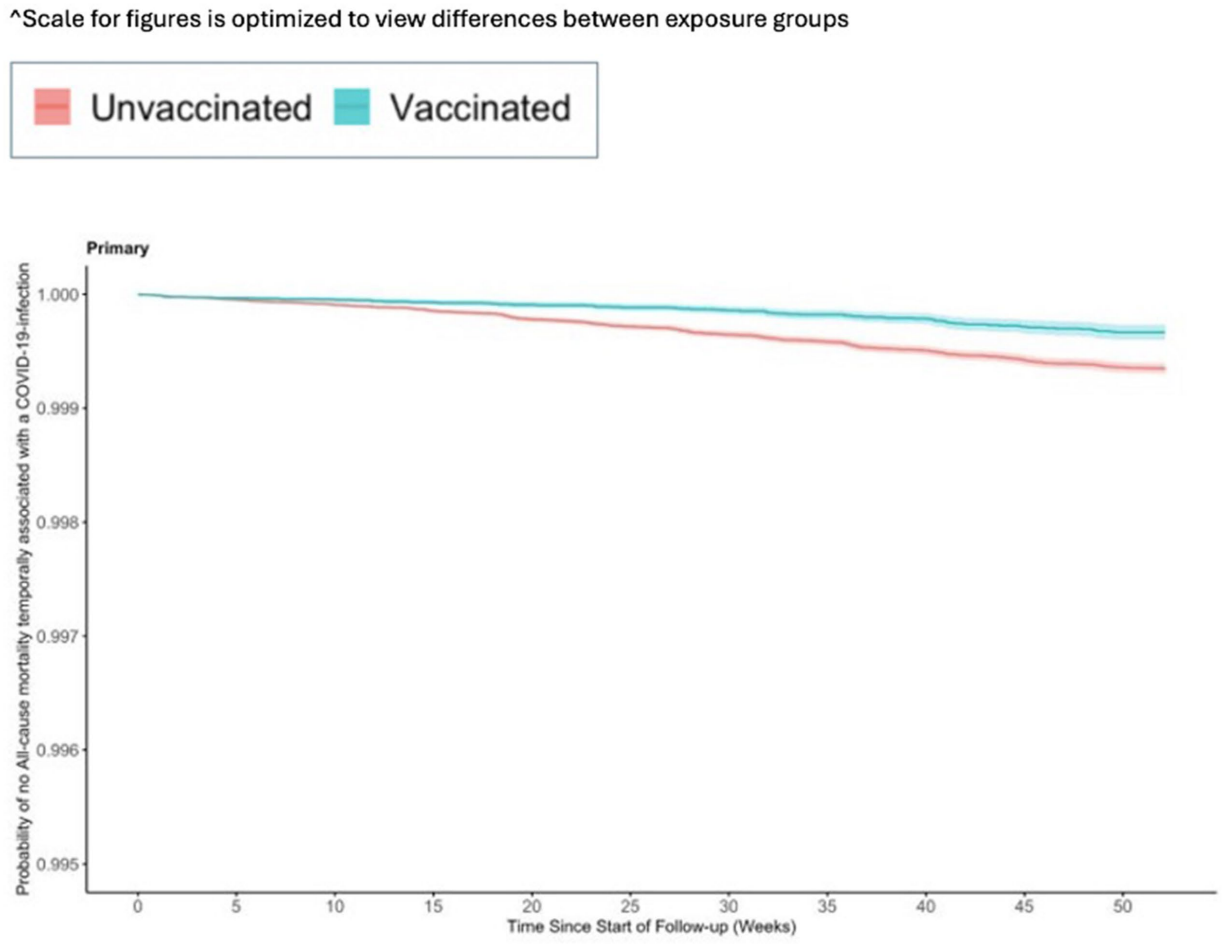
Vaccine effectiveness of the Ad26.COV2.S vaccine against all-cause mortality temporally associated with a COVID-19-infection.

**Figure 3:**
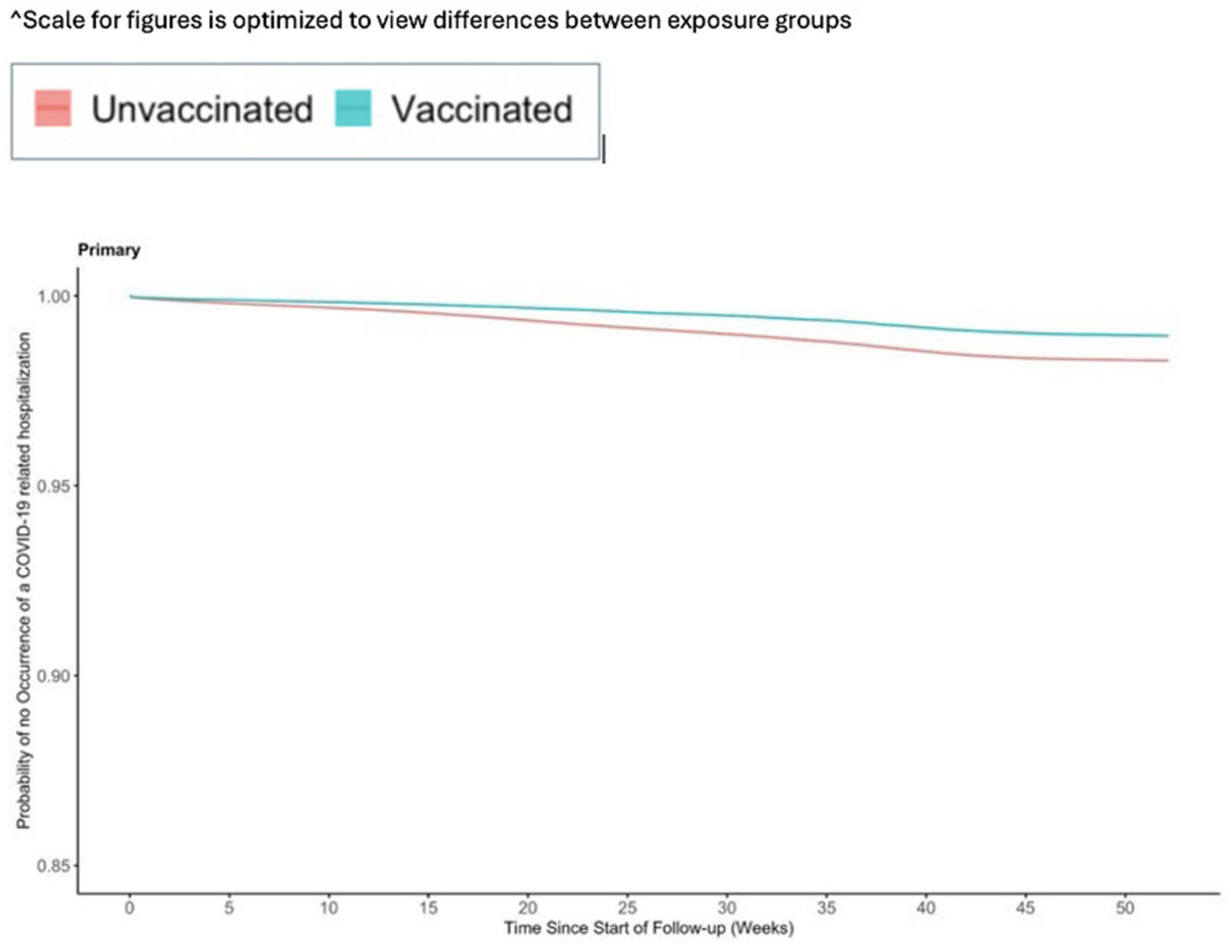
Vaccine effectiveness of the Ad26.COV2.S vaccine against any observed COVID-19 related hospitalization.

**Figure 4:**
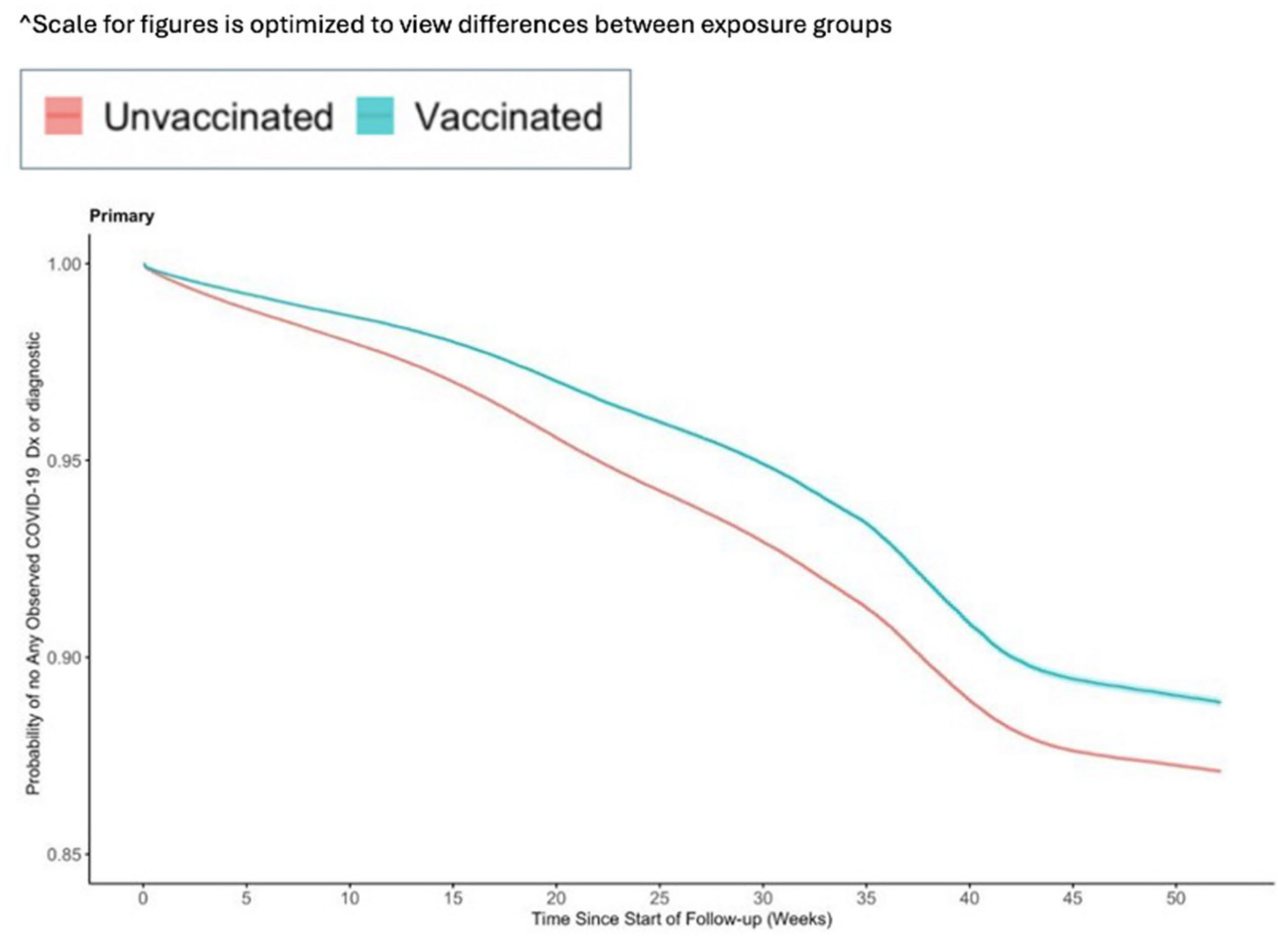
Vaccine effectiveness of the Ad26.COV2.S vaccine against any observed medically attended COVID-19.

The incidence for any observed COVID-19 was lower in the vaccinated group (110.21 per 1,000 PY) compared to the referent group (136.49 per 1,000 PY; Table 2). The incidence of COVID-19-related hospitalization was lower in the vaccinated group (10.23 per 1,000 PY) compared to the referent group (17.60 per 1,000 PY; Table 2). The incidence of all-cause mortality temporally associated with a COVID-19 disease was lower in the vaccinated group (0.30 per 1,000 PY) compared to the referent group (0.64 per 1,000 PY; Table 2). We observed the highest Ad26.COV2.S vaccine effectiveness (VE) against all-cause mortality temporally associated with a COVID-19 disease [VE: 53, 95% CI: (42, 61%), Figure 2], followed by COVID-19-related hospitalization [VE: 43, 95% CI: (40, 45%), Figure 3], and then any observed COVID-19 [VE: 20% (95% CI: 19, 21%), Figure 4; Table 2].

**Table 2:** Incidence of any observed COVID-19, COVID-19–related hospitalizations and all-cause mortality temporally associated with a COVID-19 disease, and vaccine effectiveness.

Overall Cohort	Ad26.COV2.S vaccinated group (*n* = 478,162)					PS-matched unvaccinated group (*n* = 1,897,759)					Observed VE	
	Events (*n*)	Median follow-up time (in days)	Risk per 1,000 patients	Person-years	Incidence rate (per 1,000 p-y)	Events (*n*)	Median follow-up time (in days)	Risk per 1,000 patients	Person-years	Incidence rate (per 1,000 p-y)	HR (95% CI)	VE (95% CI)
Any observed COVID-19	37,073	255 [200, 365]	77.53	336,388	110.21	159,326	243 [105, 365]	83.95	1,167,339	136.49	0.80 (0.79, 0.81)	20% (19, 21%)
COVID-19-related hospitalization	3,579	267 [209, 365]	7.48	349,828	10.23	21,601	263 [118, 365]	11.38	1,227,590	17.60	0.57 (0.55, 0.60)	43% (40, 45%)
All-cause mortality temporally associated with COVID-19 disease	107	268 [210, 365]	0.22	351,244	0.30	789	268 [120, 365]	0.42	1,236,691	0.64	0.47 (0.39, 0.58)	53% (42, 61%)

### 3.4 Vaccine effectiveness in other subgroups

We observed a higher VE against the more severe outcomes (COVID-19-related hospitalization and against all-cause mortality temporally associated with a COVID-19 disease) among individuals <65 years old compared to the overall population (Supplementary Table S2). The VE of 47% (95% CI: 45, 49%) for COVID-19-related hospitalization among individuals <65 years old was slightly higher than the VE of 43% (95% CI: 40, 45%) in the main results. Similarly, a VE of 63% (95% CI: 52, 72%) for all-cause mortality temporally associated with a COVID-19-related hospitalization among individuals <65 years old was higher than the VE of 53% (95% CI: 42, 61%) in the main results.

We evaluated subgroups of patients with comorbidities and observed lower vaccine effectiveness against COVID-19-related hospitalization within these subgroups. The VEs within subgroups ranged from 28% (95% CI: 21, 34%) among individuals with chronic neurological disorders to 38% (95% CI: 28, 47%) for those with cerebrovascular disease (Supplementary Table S2). These VEs are lower compared to the 43% VE observed against the COVID-19-related hospitalization outcome in the main results. Within subgroups, the VE against any observed COVID-19 did not vary as much as the COVID-19-related hospitalization outcome. The VE estimates for the all-cause mortality temporally associated with a COVID-19 disease for the comorbidity subgroups were lower within subgroups as well, but less precise estimates for some subgroups due to low event counts (Supplementary Table S2).

## 4 Discussion

This study evaluated the real-world effectiveness of Ad26.COV2.S against COVID-19 and related outcomes relative to no vaccination. Overall, results suggested that VE increased as the severity of COVID-19 outcomes increased. Individuals who were vaccinated with Ad26.COV2.S were at lower risk for developing COVID-19, for being hospitalized for COVID-19, and for all-cause mortality temporally associated with COVID-19 compared to unvaccinated individuals in the US. The results of subgroup analyses generally showed a similar pattern as the main analyses with VE increasing in parallel with seriousness of outcomes, albeit with lower VE in groups thought to be at higher risk of COVID-19.

Vaccine effectiveness was the highest in the subgroup analysis containing individuals under the age of 65 years, and lower in specific subgroup analyses, such as individuals over the age of 65 years and the subgroups who were deemed at risk (e.g., individuals with a history of cardiovascular disease, type 1 or type 2 diabetes, cerebrovascular disease, neurological disorders, immunosuppression, HIV, and COPD). These findings are consistent with the findings from the ENSEMBLE trial and the interim findings published from this study evaluating Ad26.COV2.S in an open-claims based COVID-19 enriched dataset (5, 6). Additionally, these findings are consistent with findings from populations outside of the US where similar patterns of decreased Ad26.COV2.S VE in older populations were seen (11). Subgroup populations deemed at risk might benefit from public health interventions and policies that appropriately address their needs such as ensuring access on time vaccination and additional booster doses.

While the overarching findings from this study are consistent with the previously published interim results, VE in this 12-month analysis is lower than that reported in the interim analysis (6). This in part reflects changes to the underlying data source and inclusion criteria. The interim results were conducted in the HealthVerity COVID-19 enriched dataset, which included only individuals who had either had a vaccination, exhibited COVID-19 symptoms or had received COVID-19 testing (6). Additionally, the interim study used both ‘open and closed’ claims, meaning individuals in the cohort did not need to have an enrollment during the baseline period or follow-up period. The rationale for these design decisions (use of the COVID-19 enriched dataset and inclusive of “open-claims”) was to enable real-time evaluation of the effectiveness of Ad26.COV2.S, using as large of a sample size as possible, a necessity given the urgent need to generate effectiveness data to help inform public health decision making (6). A limitation to this approach in the interim analysis was that the matched unvaccinated referent group was identified from individuals who had received testing for COVID-19 or exhibited COVID-19 symptoms, and not from all unvaccinated individuals in the US (6). Another limitation of the interim analysis was that individuals were not censored at the end of enrollment, which was not possible due to the open-claims data source, and therefore it is likely that individuals contributed person-time after they were no longer observable in the database. This could have resulted in a biased estimate of the vaccine effectiveness.

Of note, throughout the course of the study the COVID-19 vaccination recommendations changed to incorporate recommendations for booster vaccines and evolved to a preference for the use of mRNA vaccines in the US. While this study only focuses on a single Ad26.COV2.S dose and does not account for boosters or compare the single Ad26.COV2.S vaccine to mRNA vaccines, this study provides evidence for the use of Ad26.COV2.S as an alternative to preventing COVID-19 especially in unvaccinated populations.

### 4.1 Limitations

As is the limitation of all findings from claims data, the findings from this study relied on the accuracy of diagnosis, medication and procedure codes to identify individuals and treatments, as well as to evaluate characteristics and outcomes. These measures may be influenced by miscoding or inaccuracies in the data. Also, there might have been instances of exposure misclassification bias in both the vaccinated and unvaccinated cohorts, in which additional doses of Ad26.COV2.S or other vaccines were administered but not recorded. Individuals may not seek care for non-serious outcomes, including mild COVID-19 symptoms leading to milder outcomes not being fully recorded in the claims data. Additionally, as home testing became available these results would not be recorded for those not seeking care including potentially missing milder cases of breakthrough disease.

The Health Verity dataset is primarily insurance-based, therefore generalizability may be limited to the insured US population and may not fully capture individuals in lower socioeconomic brackets or the uninsured, as these groups may interact with the healthcare system differently. This might have introduced some bias in the study’s findings. In addition, some clinical measures related to likelihood of vaccination and risk factors associated with severity of disease are not directly observable in claims data. Still, it is expected that controlling for demographics, baseline interaction with the healthcare system and many comorbidities mitigated residual confounding.

In the HealthVerity dataset, the COVID-19 antibody tests do not capture the granularity of specific test targets (nucleocapsid vs. spike). As a result, it was not possible to differentiate between individuals with prior COVID-19 and prior vaccination therefore, a subset of individuals with a previous COVID-19 vaccination may have been captured even if they had tested positive for COVID-19. To mitigate the impact of this observation, approximately 90% of individuals testing positive for COVID-19 in the HealthVerity dataset were identified by diagnostic NAAT laboratory tests.

## 5 Conclusion

This population-representative cohort study in U.S. clinical practice showed that a single dose of Ad26.COV2.S is effective for at least 12 months against several COVID-19 related outcomes. Individuals who were vaccinated with Ad26.COV2.S were at lower risk for developing COVID-19, for being hospitalized for COVID-19, and for all-cause mortality temporally associated with COVID-19 compared to unvaccinated individuals in the U.S during Alpha, Delta, and Omicron BA.1, BA.2, BA.212.1, and BA.5 variants circulation.

## Data Availability

The raw data supporting the conclusions of this article will be made available by the authors, without undue reservation.
