# Supplementary material for: An observational post-authorization study to assess the effectiveness of a single dose Ad26.COV2.S for the prevention of COVID-19 using real-world data
Source: Front Public Health. 2024 Dec 4;12:1501919. doi: 10.3389/fpubh.2024.1501919 (PMC11652512; doi:10.3389/fpubh.2024.1501919)

Supplementary Material

# Table 1. Variable Definitions

| **Variable** | **Definition** |
| --- | --- |
| **Primary Outcomes** | |
| Any observed COVID-19 | Positive COVID-19 diagnostic test (NAAT) result in laboratory data  OR  Any medical claim, inpatient hospital encounter or outpatient hospital encounter indicating diagnosis of COVID-19 as listed in ANNEX 1 of the protocol. |
| Occurrence of a COVID-19-related hospitalization | Any inpatient medical claim or inpatient hospital encounter for which any observed COVID-19 began in the 21 days prior to the start of the hospitalization through the last day of the hospitalization. |
| All-cause mortality during a COVID-19-related hospitalization  Not analyzed in the early analysis. | Any death observed during an inpatient hospital encounter for which any observed COVID-19 began in the 21 days prior to the start of the hospitalization through the last day of the hospitalization. The definition of death is reported in ANNEX 1 of the protocol. |
| **Pre-exposure Covariates Used for Confounding Adjustment via Propensity Score Matching** | |
| **Variable Name** | **Real-World Data Definition** |
| Age | Age defined as continuous numeric variable for propensity score  Modeling in the data |
| Sex | Male/Female as defined in the data |
| Gagne Comorbidity Score | Gagne Combined Comorbidity Index |
| Insurance Type | Medicaid/Medicare/Commercial |
| Chronic pulmonary diseases | Any medical claim, inpatient hospital encounter, or outpatient hospital encounter with one of the following ICD-10-CM codes: I27.8, I27.9, J40, J41, J42, J43, J44, J45, J46, J47, J60, J61, J62, J63, J64, J65, J66, J67, J68.4, J70.1, J70.3 |
| Liver diseases | Any medical claim, inpatient hospital encounter, or outpatient hospital encounter with one of the following ICD-10-CM codes: B18, I85, I86.4, I98.2, K70, K71.1, K71.3, K71.4, K71.5, K71.7, K72, K73, K74, K76.0, K76.2, K76.3, K76.4, K76.5, K76.6, K76.7, K76.8, K76.9, Z94.4 |
| Renal diseases | Any medical claim, inpatient hospital encounter, or outpatient hospital encounter with one of the following ICD-10-CM codes: I12.0, 13.1, N03.2, N03.3, N03.4, N03.5, N03.6, N03.7, N05.2, N05.3, N05.4, N05.5, N05.6, N05.7, N18, N19, N25.0, Z49.0, Z49.1, Z49.2, Z94.0, Z99.2 |
| Diabetes  (Type 1 or 2) | Any medical claim, inpatient hospital encounter, or outpatient hospital encounter with one of the following ICD-10-CM codes: E10, E11, E12, E13, E14 |
| Hypertension | Any medical claim, inpatient hospital encounter, or outpatient hospital encounter with one of the following ICD-10-CM codes: I10, I11, I12, I13, I15 |
| Obesity | Any medical claim, inpatient hospital encounter, or outpatient hospital encounter with one of the following ICD-10-CM codes: Z68.30, Z68.31, Z68.32, Z68.33, Z68.34, Z68.35, Z68.36, Z68.37, Z68.38, Z68.39, Z68.41, Z68.42, Z68.43, Z68.44, Z68.45, E66 |
| Malignancy  (excluding of the skin) | Any medical claim, inpatient hospital encounter, or outpatient hospital encounter with one of the following ICD-10-CM codes: C00, C01, C02, C03, C04, C05, C06, C07, C08, C09, C10, C11, C12, C13, C14, C15, C16, C17, C18, C19, C20, C21, C22, C23, C24, C25, C26, C30, C31, C32, C33, C34, C37, C38, C39, C40, C41, C43, C45, C46, C47, C48, C49, C50, C51, C52, C53, C54, C55, C56, C57, C58, C60, C61, C62, C63, C64, C65, C66, C67, C68, C69, C70, C71, C72, C73, C74, C75, C76, C81, C82, C83, C84, C85, C88, C90, C91, C92, C93, C94, C95, C96, C97 |
| Congestive Heart Failure | Any medical claim, inpatient hospital encounter, or outpatient hospital encounter with one of the following ICD-10-CM codes: 18.84, I13.0, I13.2, I25.5, I42.1, I42.3, I42.6, I42.8, I43, I50.1, I50.2, I50.20, I50.21, I50.22, I50.23, I50.3, I50.30, I50.31, I50.33, I50.4, I50.40, I50.41, I50.42, I50.43, P29.0, I09.9, I11.0, I42, I42.0, I42.2, I42.4, I42.5, I42.7, I42.9, I50, I50.32, I50.9, I51.7 |
| Cardiac Arrhythmias | Any medical claim, inpatient hospital encounter, or outpatient hospital encounter with one of the following ICD-10-CM codes: I44.0, I44.1, I44.3, I44.30, I44.39, I44.4, I44.5, I44.6, I44.60, I44.69, I44.7, I45.0, I45.1, I45.10, I45.19, I45.2, I45.4, I45.5, I45.6, I45.8, I45.81, I45.89, I45.9, I47, I47.0, I47.1, I47.2, I47.9, I48, I48.0, I48.1, I48.2, I48.3, I48.4, I48.9, I48.91, I48.92, I49, I49.0, I49.01, I49.02, I49.1, I49.2, I49.3, I49.4, I49.40, I49.49, I49.5, I49.8, I49.9, R00.0, R00.1, R00.8, T82.1, T82.11, T82.110, T82.110A, T82.110D, T82.110S, T82.111, T82.111A, T82.111D, T82.111S, T82.118, T82.118A, T82.118D, T82.118S, T82.119, T82.119A, T82.119D, T82.119S, T82.12, T82.120, T82.120A, T82.120D, T82.120S, T82.121, T82.121A, T82.121D, T82.121S, T82.128, T82.128A, T82.128D, T82.128S, T82.129, T82.129A, T82.129D, T82.129S, T82.19, T82.190, T82.190A, T82.190D, T82.190S, T82.191, T82.191A, T82.191D, T82.191S, T82.198, T82.198A, T82.198D, T82.198S, T82.199, T82.199A, T82.199D, T82.199S, Z45.0, Z45.01, Z45.010, Z45.018, Z45.02, Z45.09, Z95.0, Z95.810, Z95.818, Z95.9 |
| Coagulopathy | Any medical claim, inpatient hospital encounter, or outpatient hospital encounter with one of the following ICD-10-CM codes: D65, D66, D67, D68, D68.0, D68.1, D68.2, D68.3, D68.31, D68.311, D68.312, D68.318, D68.32, D68.4, D68.5, D68.51, D68.52, D68.59, D68.6, D68.61, D68.62, D68.69, D68.8, D68.9, D69.1, D69.3, D69.4, D69.41, D69.42, D69.49, D69.5, D69.51, D69.59, D69.6 |
| Peripheral Vascular Disorder | Any medical claim, inpatient hospital encounter, or outpatient hospital encounter with one of the following ICD-10-CM codes: E08.51, E08.52, E09.51, E09.52, E10.51, E10.52, E11.51, E13.51, E13.52, I67.0, I70, I70.0, I70.1, I70.2, I70.20, I70.201, I70.202, I70.203, I70.208, I70.209, I70.21, I70.211, I70.212, I70.213, I70.218, I70.219, I70.22, I70.221, I70.222, I70.223, I70.228, I70.229, I70.23, I70.231, I70.232, I70.233, I70.234, I70.235, I70.238, I70.239, I70.24, I70.241, I70.242, I70.243, I70.244, I70.245, I70.248, I70.249, I70.25, I70.26, I70.261, I70.262, I70.263, I70.268, I70.269, I70.29, I70.291, I70.292, I70.293, I70.298, I70.299, I70.3, I70.30, I70.301, I70.302, I70.303, I70.308, I70.309, I70.31, I70.311, I70.312, I70.313, I70.318, I70.319, I70.32, I70.321, I70.322, I70.323, I70.328, I70.329, I70.33, I70.331, I70.332, I70.333, I70.334, I70.335, I70.338, I70.339, I70.34, I70.341, I70.342, I70.343, I70.344, I70.345, I70.348, I70.349, I70.35, I70.36, I70.361, I70.362, I70.363, I70.368, I70.369, I70.39, I70.391, I70.392, I70.393, I70.398, I70.399, I70.4, I70.40, I70.401, I70.402, I70.403, I70.408, I70.409, I70.41, I70.411, I70.412, I70.413, I70.418, I70.419, I70.42, I70.421, I70.422, I70.423, I70.428, I70.429, I70.43, I70.431, I70.432, I70.433, I70.434, I70.435, I70.438, I70.439, I70.44, I70.441, I70.442, I70.443, I70.444, I70.445, I70.448, I70.449, I70.45, I70.46, I70.461, I70.462, I70.463, I70.468, I70.469, I70.49, I70.491, I70.492, I70.493, I70.498, I70.499, I70.5, I70.50, I70.501, I70.502, I70.503, I70.508, I70.509, I70.51, I70.511, I70.512, I70.513, I70.518, I70.519, I70.52, I70.521, I70.522, I70.523, I70.528, I70.529, I70.53, I70.531, I70.532, I70.533, I70.534, I70.535, I70.538, I70.539, I70.54, I70.541, I70.542, I70.543, I70.544, I70.545, I70.548, I70.549, I70.55, I70.56, I70.561, I70.562, I70.563, I70.568, I70.569, I70.59, I70.591, I70.592, I70.593, I70.598, I70.599, I70.6, I70.60, I70.601, I70.602, I70.603, I70.608, I70.609, I70.61, I70.611, I70.612, I70.613, I70.618, I70.619, I70.62, I70.621, I70.622, I70.623, I70.628, I70.629, I70.63, I70.631, I70.632, I70.633, I70.634, I70.635, I70.638, I70.639, I70.64, I70.641, I70.642, I70.643, I70.644, I70.645, I70.648, I70.649, I70.65, I70.66, I70.661, I70.662, I70.663, I70.668, I70.669, I70.69, I70.691, I70.692, I70.693, I70.698, I70.699, I70.7, I70.70, I70.701, I70.702, I70.703, I70.708, I70.709, I70.71, I70.711, I70.712, I70.713, I70.718, I70.719, I70.72, I70.721, I70.722, I70.723, I70.728, I70.729, I70.73, I70.731, I70.732, I70.733, I70.734, I70.735, I70.738, I70.739, I70.74, I70.741, I70.742, I70.743, I70.744, I70.745, I70.748, I70.749, I70.75, I70.76, I70.761, I70.762, I70.763, I70.768, I70.769, I70.79, I70.791, I70.792, I70.793, I70.798, I70.799, I70.8, I70.9, I70.90, I70.91, I70.92, I71, I71.0, I71.00, I71.01, I71.02, I71.03, I71.1, I71.2, I71.3, I71.4, I71.5, I71.6, I71.8, I71.9, I73.1, I73.8, I73.81, I73.89, I73.9, I77.1, I77.71, I77.72, I77.73, I77.74, I77.79, I79, I79.0, I79.1, I79.8, K55.1, K55.8, K55.9, Z95.82, Z95.820, Z95.828, Z95.9 |
| Pulmonary Circulation Disorders | Any medical claim, inpatient hospital encounter, or outpatient hospital encounter with one of the following ICD-10-CM codes: I26, I26.0, I26.01, I26.02, I26.09, I26.9, I26.90, I26.92, I26.99, I27, I27.0, I27.1, I27.2, I27.8, I27.81, I27.82, I27.89, I27.9, I28.0, I28.8, I28.9 |
| Cerebrovascular Disease | Any medical claim, inpatient hospital encounter, or outpatient hospital encounter with one of the following ICD-10-CM codes: G45, G45.0, G45.1, G45.2, G45.3, G45.4, G45.8, G45.9, I60, I60.0, I60.00, I60.01, I60.02, I60.1, I60.10, I60.11, I60.12, I60.2, I60.20, I60.21, I60.22, I60.3, I60.30, I60.31, I60.32, I60.4, I60.5, I60.50, I60.51, I60.52, I60.6, I60.7, I60.8, I60.9, I61, I61.0, I61.1, I61.2, I61.3, I61.4, I61.5, I61.6, I61.8, I61.9, I62, I62.0, I62.00, I62.01, I62.02, I62.03, I62.1, I62.9, I63, I63.0, I63.00, I63.01, I63.011, I63.012, I63.013, I63.019, I63.02, I63.03, I63.031, I63.032, I63.033, I63.039, I63.09, I63.1, I63.10, I63.11, I63.111, I63.112, I63.113, I63.119, I63.12, I63.13, I63.131, I63.132, I63.133, I63.139, I63.19, I63.2, I63.20, I63.21, I63.211, I63.212, I63.213, I63.219, I63.22, I63.23, I63.231, I63.232, I63.233, I63.239, I63.29, I63.3, I63.30, I63.31, I63.311, I63.312, I63.313, I63.319, I63.32, I63.321, I63.322, I63.323, I63.329, I63.33, I63.331, I63.332, I63.333, I63.339, I63.34, I63.341, I63.342, I63.343, I63.349, I63.39, I63.4, I63.40, I63.41, I63.411, I63.412, I63.413, I63.419, I63.42, I63.421, I63.422, I63.423, I63.429, I63.43, I63.431, I63.432, I63.433, I63.439, I63.44, I63.441, I63.442, I63.443, I63.449, I63.49, I63.5, I63.50, I63.51, I63.511, I63.512, I63.513, I63.519, I63.52, I63.521, I63.522, I63.523, I63.529, I63.53, I63.531, I63.532, I63.533, I63.539, I63.54, I63.541, I63.542, I63.543, I63.549, I63.59, I63.6, I63.8, I63.81, I63.89, I63.9 |
| Any Cardiovascular Disease | Any medical claim, inpatient hospital encounter, or outpatient hospital encounter with one of the following ICD-10-CM codes: I01, I01.0, I01.9, I02.0, I05.2, I05.9, I06.0, I06.8, I06.9, I07, I07.0, I07.1, I08.0, I08.1, I08.9, I09, I09.1, I09.2, I09.89, I11, I11.0, I11.9, I12, I12.0, I13, I13.1, I13.10, I13.11, I15, I15.8, I15.9, I16, I16.1, I20, I20.0, I21, I21.01, I21.02, I21.09, I21.1, I21.11, I21.19, I21.A, I22.8, I22.9, I23.1, I23.4, I23.5, I23.6, I23.7, I23.8, I24.0, I24.1, I24.9, I25.1, I25.11, I25.110, I25.111, I25.2, I25.4, I25.41, I25.6, I25.7, I25.700, I25.710, I25.720, I25.721, I25.728, I25.731, I25.738, I25.739, I25.750, I25.76, I25.760, I25.769, I25.790, I25.799, I25.8, I25.81, I25.810, I25.82, I25.89, I25.9, I26.01, I26.02, I26.9, I27.1, I27.20, I27.21, I27.23, I27.29, I27.83, I28.0, I28.1, I28.8, I28.9, I30.0, I31, I31.0, I31.1, I31.9, I32, I33.9, I34, I34.1, I34.8, I34.9, I35.8, I35.9, I36.1, I36.2, I36.8, I37, I37.0, I37.1, I37.8, I40, I40.0, I40.1, I42, I42.0, I42.1, I42.3, I42.5, I42.9, I43, I44, I44.0, I44.1, I44.2, I44.5, I44.6, I44.60, I44.69, I45, I45.0, I45.10, I45.19, I45.6, I45.9, I46.2, I46.8, I47.0, I47.1, I47.9, I48, I48.3, I48.9, I48.92, I49.0, I49.1, I49.2, I49.40, I49.8, I49.9, I50, I50.3, I50.30, I50.31, I50.33, I50.42, I50.8, I50.811, I51, I51.0, I51.1, I51.81, I51.89, I52, I60, I60.00, I60.02, I60.12, I60.2, I60.20, I60.21, I60.22, I60.30, I60.50, I60.51, I60.6, I60.7, I60.8, I60.9, I61.2, I61.4, I61.5, I61.6, I62, I62.0, I62.00, I62.01, I62.02, I62.1, I62.9, I63, I63.01, I63.012, I63.013, I63.019, I63.03, I63.033, I63.039, I63.09, I63.10, I63.11, I63.111, I63.112, I63.113, I63.119, I63.13, I63.131, I63.132, I63.133, I63.139, I63.19, I63.212, I63.213, I63.219, I63.23, I63.231, I63.232, I63.29, I63.30, I63.31, I63.312, I63.319, I63.32, I63.321, I63.33, I63.331, I63.332, I63.39, I63.413, I63.42, I63.421, I63.423, I63.43, I63.431, I63.433, I63.442, I63.449, I63.5, I63.512, I63.513, I63.522, I63.529, I63.531, I63.533, I63.54, I63.59, I63.81, I63.89, I63.9, I65, I65.0, I65.01, I65.02, I65.03, I65.21, I65.23, I65.8, I66, I66.0, I66.01, I66.03, I66.09, I66.1, I66.13, I66.22, I66.29, I66.8, I66.9, I67.1, I67.3, I67.5, I67.8, I67.81, I67.84, I67.841, I67.858, I67.9, I68, I68.2, I69, I69.0, I69.01, I69.011, I69.012, I69.013, I69.014, I69.015, I69.018, I69.019, I69.020, I69.022, I69.023, I69.028, I69.039, I69.042, I69.049, I69.05, I69.051, I69.06, I69.061, I69.065, I69.069, I69.09, I69.091, I69.11, I69.112, I69.113, I69.118, I69.119, I69.12, I69.120, I69.122, I69.13, I69.133, I69.14, I69.142, I69.15, I69.151, I69.152, I69.153, I69.159, I69.161, I69.162, I69.163, I69.165, I69.169, I69.19, I69.190, I69.193, I69.198, I69.20, I69.210, I69.211, I69.212, I69.218, I69.220, I69.221, I69.222, I69.228, I69.23, I69.233, I69.234, I69.239, I69.241, I69.25, I69.251, I69.253, I69.254, I69.259, I69.265, I69.269, I69.290, I69.292, I69.293, I69.298, I69.3, I69.31, I69.310, I69.311, I69.312, I69.313, I69.314, I69.315, I69.318, I69.319, I69.32, I69.328, I69.331, I69.333, I69.339, I69.34, I69.344, I69.35, I69.351, I69.352, I69.36, I69.361, I69.365, I69.39, I69.390, I69.392, I69.393, I69.810, I69.811, I69.814, I69.818, I69.819, I69.82, I69.823, I69.83, I69.832, I69.84, I69.841, I69.842, I69.851, I69.86, I69.862, I69.863, I69.865, I69.9, I69.910, I69.912, I69.913, I69.914, I69.923, I69.93, I69.933, I69.934, I69.939, I69.941, I69.942, I69.951, I69.952, I69.953, I69.954, I69.96, I69.961, I69.962, I69.990, I69.991, I69.992, I70.1, I70.20, I70.201, I70.202, I70.203, I70.208, I70.21, I70.212, I70.219, I70.22, I70.221, I70.223, I70.229, I70.234, I70.235, I70.24, I70.241, I70.243, I70.245, I70.248, I70.261, I70.268, I70.269, I70.29, I70.292, I70.293, I70.299, I70.3, I70.30, I70.309, I70.31, I70.311, I70.312, I70.318, I70.323, I70.334, I70.338, I70.34, I70.341, I70.342, I70.343, I70.345, I70.348, I70.36, I70.361, I70.363, I70.368, I70.392, I70.399, I70.4, I70.40, I70.402, I70.403, I70.408, I70.413, I70.418, I70.419, I70.422, I70.429, I70.43, I70.438, I70.439, I70.44, I70.442, I70.444, I70.448, I70.45, I70.461, I70.463, I70.49, I70.491, I70.492, I70.498, I70.50, I70.502, I70.51, I70.511, I70.512, I70.519, I70.521, I70.522, I70.523, I70.528, I70.529, I70.53, I70.531, I70.532, I70.533, I70.534, I70.535, I70.543, I70.544, I70.549, I70.55, I70.563, I70.568, I70.569, I70.59, I70.591, I70.592, I70.593, I70.60, I70.601, I70.602, I70.61, I70.611, I70.612, I70.618, I70.619, I70.621, I70.622, I70.623, I70.628, I70.63, I70.633, I70.634, I70.635, I70.638, I70.64, I70.642, I70.643, I70.644, I70.648, I70.65, I70.662, I70.663, I70.69, I70.691, I70.698, I70.7, I70.70, I70.701, I70.702, I70.703, I70.708, I70.71, I70.711, I70.713, I70.721, I70.723, I70.73, I70.732, I70.735, I70.738, I70.74, I70.742, I70.743, I70.748, I70.749, I70.75, I70.76, I70.763, I70.768, I70.769, I70.792, I70.8, I70.91, I70.92, I71.00, I71.01, I71.02, I71.03, I71.3, I72, I72.0, I72.2, I72.5, I72.8, I72.9, I73, I73.0, I73.00, I73.01, I73.8, I73.81, I73.89, I74.0, I74.09, I74.10, I74.19, I74.2, I74.3, I74.5, I75, I75.0, I75.01, I75.011, I75.012, I75.013, I75.02, I75.021, I75.8, I75.81, I75.89, I76, I77.0, I77.1, I77.3, I77.5, I77.71, I77.74, I77.75, I77.76, I77.77, I77.79, I77.812, I77.819, I77.89, I78, I78.8, I78.9, I79, I79.0, I79.1, I80, I80.01, I80.02, I80.10, I80.20, I80.209, I80.21, I80.211, I80.219, I80.22, I80.221, I80.222, I80.229, I80.231, I80.232, I80.292, I80.8, I81, I82.1, I82.2, I82.21, I82.220, I82.221, I82.29, I82.290, I82.291, I82.4, I82.40, I82.401, I82.403, I82.411, I82.421, I82.422, I82.429, I82.431, I82.432, I82.433, I82.44, I82.441, I82.442, I82.443, I82.49, I82.499, I82.4Y, I82.4Y1, I82.4Y2, I82.4Y9, I82.4Z2, I82.4Z9, I82.50, I82.501, I82.502, I82.509, I82.51, I82.512, I82.521, I82.53, I82.531, I82.533, I82.539, I82.54, I82.541, I82.543, I82.549, I82.591, I82.5Y1, I82.5Y9, I82.5Z, I82.602, I82.609, I82.613, I82.62, I82.629, I82.7, I82.70, I82.702, I82.703, I82.709, I82.71, I82.712, I82.72, I82.721, I82.811, I82.813, I82.89, I82.890, I82.90, I82.91, I82.A12, I82.A2, I82.A21, I82.A22, I82.A29, I82.B12, I82.B13, I82.B19, I82.B2, I82.B22, I82.B23, I82.C, I82.C12, I82.C13, I82.C19, I82.C21, I82.C22, I82.C23, I83, I83.00, I83.002, I83.003, I83.005, I83.009, I83.01, I83.011, I83.013, I83.014, I83.019, I83.02, I83.022, I83.023, I83.025, I83.028, I83.1, I83.12, I83.2, I83.201, I83.204, I83.21, I83.213, I83.214, I83.215, I83.219, I83.222, I83.223, I83.225, I83.811, I83.812, I83.819, I83.891, I83.892, I83.899, I83.9, I83.90, I85.0, I85.00, I85.11, I86, I86.1, I86.2, I87.00, I87.002, I87.009, I87.01, I87.011, I87.02, I87.03, I87.031, I87.09, I87.091, I87.092, I87.1, I87.30, I87.301, I87.303, I87.309, I87.31, I87.311, I87.312, I87.319, I87.32, I87.321, I87.322, I87.323, I87.33, I87.331, I87.332, I87.339, I87.392, I87.393, I87.8, I88.0, I88.1, I88.8, I89, I89.1, I95.2, I95.3, I95.89, I96, I97.1, I97.120, I97.19, I97.191, I97.3, I97.411, I97.42, I97.52, I97.610, I97.618, I97.62, I97.620, I97.621, I97.622, I97.631, I97.638, I97.641, I97.648, I97.7, I97.79, I97.790, I97.81, I97.810, I97.82, I97.821, I97.89, I99, I00, I01.1, I01.2, I01.8, I02, I02.9, I05, I05.0, I05.1, I05.8, I06, I06.1, I06.2, I07.2, I07.8, I07.9, I08, I08.2, I08.3, I08.8, I09.0, I09.8, I09.81, I09.9, I10, I12.9, I13.0, I13.2, I15.0, I15.1, I15.2, I16.0, I16.9, I20.1, I20.8, I20.9, I21.0, I21.2, I21.21, I21.29, I21.3, I21.4, I21.9, I21.A1, I21.A9, I22, I22.0, I22.1, I22.2, I23, I23.0, I23.2, I23.3, I24, I24.8, I25, I25.10, I25.118, I25.119, I25.3, I25.42, I25.5, I25.70, I25.701, I25.708, I25.709, I25.71, I25.711, I25.718, I25.719, I25.72, I25.729, I25.73, I25.730, I25.75, I25.751, I25.758, I25.759, I25.761, I25.768, I25.79, I25.791, I25.798, I25.811, I25.812, I25.83, I25.84, I26, I26.0, I26.09, I26.90, I26.92, I26.99, I27, I27.0, I27.2, I27.22, I27.24, I27.8, I27.81, I27.82, I27.89, I27.9, I28, I30, I30.1, I30.8, I30.9, I31.2, I31.3, I31.4, I31.8, I33, I33.0, I34.0, I34.2, I35, I35.0, I35.1, I35.2, I36, I36.0, I36.9, I37.2, I37.9, I38, I39, I40.8, I40.9, I41, I42.2, I42.4, I42.6, I42.7, I42.8, I44.3, I44.30, I44.39, I44.4, I44.7, I45.1, I45.2, I45.3, I45.4, I45.5, I45.8, I45.81, I45.89, I46, I46.9, I47, I47.2, I48.0, I48.1, I48.2, I48.4, I48.91, I49, I49.01, I49.02, I49.3, I49.4, I49.49, I49.5, I50.1, I50.2, I50.20, I50.21, I50.22, I50.23, I50.32, I50.4, I50.40, I50.41, I50.43, I50.81, I50.810, I50.812, I50.813, I50.814, I50.82, I50.83, I50.84, I50.89, I50.9, I51.2, I51.3, I51.4, I51.5, I51.7, I51.8, I51.9, I60.0, I60.01, I60.1, I60.10, I60.11, I60.3, I60.31, I60.32, I60.4, I60.5, I60.52, I61, I61.0, I61.1, I61.3, I61.8, I61.9, I62.03, I63.0, I63.00, I63.011, I63.02, I63.031, I63.032, I63.1, I63.12, I63.2, I63.20, I63.21, I63.211, I63.22, I63.233, I63.239, I63.3, I63.311, I63.313, I63.322, I63.323, I63.329, I63.333, I63.339, I63.34, I63.341, I63.342, I63.343, I63.349, I63.4, I63.40, I63.41, I63.411, I63.412, I63.419, I63.422, I63.429, I63.432, I63.439, I63.44, I63.441, I63.443, I63.49, I63.50, I63.51, I63.511, I63.519, I63.52, I63.521, I63.523, I63.53, I63.532, I63.539, I63.541, I63.542, I63.543, I63.549, I63.6, I63.8, I65.09, I65.1, I65.2, I65.22, I65.29, I65.9, I66.02, I66.11, I66.12, I66.19, I66.2, I66.21, I66.23, I66.3, I67, I67.0, I67.2, I67.4, I67.6, I67.7, I67.82, I67.83, I67.848, I67.85, I67.850, I67.89, I68.0, I68.8, I69.00, I69.010, I69.02, I69.021, I69.03, I69.031, I69.032, I69.033, I69.034, I69.04, I69.041, I69.043, I69.044, I69.052, I69.053, I69.054, I69.059, I69.062, I69.063, I69.064, I69.090, I69.092, I69.093, I69.098, I69.1, I69.10, I69.110, I69.111, I69.114, I69.115, I69.121, I69.123, I69.128, I69.131, I69.132, I69.134, I69.139, I69.141, I69.143, I69.144, I69.149, I69.154, I69.16, I69.164, I69.191, I69.192, I69.2, I69.21, I69.213, I69.214, I69.215, I69.219, I69.22, I69.223, I69.231, I69.232, I69.24, I69.242, I69.243, I69.244, I69.249, I69.252, I69.26, I69.261, I69.262, I69.263, I69.264, I69.29, I69.291, I69.30, I69.320, I69.321, I69.322, I69.323, I69.33, I69.332, I69.334, I69.341, I69.342, I69.343, I69.349, I69.353, I69.354, I69.359, I69.362, I69.363, I69.364, I69.369, I69.391, I69.398, I69.8, I69.80, I69.81, I69.812, I69.813, I69.815, I69.820, I69.821, I69.822, I69.828, I69.831, I69.833, I69.834, I69.839, I69.843, I69.844, I69.849, I69.85, I69.852, I69.853, I69.854, I69.859, I69.861, I69.864, I69.869, I69.89, I69.890, I69.891, I69.892, I69.893, I69.898, I69.90, I69.91, I69.911, I69.915, I69.918, I69.919, I69.92, I69.920, I69.921, I69.922, I69.928, I69.931, I69.932, I69.94, I69.943, I69.944, I69.949, I69.95, I69.959, I69.963, I69.964, I69.965, I69.969, I69.99, I69.993, I69.998, I70, I70.0, I70.2, I70.209, I70.211, I70.213, I70.218, I70.222, I70.228, I70.23, I70.231, I70.232, I70.233, I70.238, I70.239, I70.242, I70.244, I70.249, I70.25, I70.26, I70.262, I70.263, I70.291, I70.298, I70.301, I70.302, I70.303, I70.308, I70.313, I70.319, I70.32, I70.321, I70.322, I70.328, I70.329, I70.33, I70.331, I70.332, I70.333, I70.335, I70.339, I70.344, I70.349, I70.35, I70.362, I70.369, I70.39, I70.391, I70.393, I70.398, I70.401, I70.409, I70.41, I70.411, I70.412, I70.42, I70.421, I70.423, I70.428, I70.431, I70.432, I70.433, I70.434, I70.435, I70.441, I70.443, I70.445, I70.449, I70.46, I70.462, I70.468, I70.469, I70.493, I70.499, I70.5, I70.501, I70.503, I70.508, I70.509, I70.513, I70.518, I70.52, I70.538, I70.539, I70.54, I70.541, I70.542, I70.545, I70.548, I70.56, I70.561, I70.562, I70.598, I70.599, I70.6, I70.603, I70.608, I70.609, I70.613, I70.62, I70.629, I70.631, I70.632, I70.639, I70.641, I70.645, I70.649, I70.66, I70.661, I70.668, I70.669, I70.692, I70.693, I70.699, I70.709, I70.712, I70.718, I70.719, I70.72, I70.722, I70.728, I70.729, I70.731, I70.733, I70.734, I70.739, I70.741, I70.744, I70.745, I70.761, I70.762, I70.79, I70.791, I70.793, I70.798, I70.799, I70.9, I70.90, I71, I71.0, I71.1, I71.2, I71.4, I71.5, I71.6, I71.8, I71.9, I72.1, I72.3, I72.4, I72.6, I73.1, I73.9, I74, I74.01, I74.1, I74.11, I74.4, I74.8, I74.9, I75.019, I75.022, I75.023, I75.029, I77, I77.2, I77.4, I77.6, I77.7, I77.70, I77.72, I77.73, I77.8, I77.81, I77.810, I77.811, I77.9, I78.0, I78.1, I79.8, I80.0, I80.00, I80.03, I80.1, I80.11, I80.12, I80.13, I80.2, I80.201, I80.202, I80.203, I80.212, I80.213, I80.223, I80.23, I80.233, I80.239, I80.29, I80.291, I80.293, I80.299, I80.3, I80.9, I82, I82.0, I82.210, I82.211, I82.22, I82.3, I82.402, I82.409, I82.41, I82.412, I82.413, I82.419, I82.42, I82.423, I82.43, I82.439, I82.449, I82.491, I82.492, I82.493, I82.4Y3, I82.4Z, I82.4Z1, I82.4Z3, I82.5, I82.503, I82.511, I82.513, I82.519, I82.52, I82.522, I82.523, I82.529, I82.532, I82.542, I82.59, I82.592, I82.593, I82.599, I82.5Y, I82.5Y2, I82.5Y3, I82.5Z1, I82.5Z2, I82.5Z3, I82.5Z9, I82.6, I82.60, I82.601, I82.603, I82.61, I82.611, I82.612, I82.619, I82.621, I82.622, I82.623, I82.701, I82.711, I82.713, I82.719, I82.722, I82.723, I82.729, I82.8, I82.81, I82.812, I82.819, I82.891, I82.9, I82.A, I82.A1, I82.A11, I82.A13, I82.A19, I82.A23, I82.B, I82.B1, I82.B11, I82.B21, I82.B29, I82.C1, I82.C11, I82.C2, I82.C29, I83.0, I83.001, I83.004, I83.008, I83.012, I83.015, I83.018, I83.021, I83.024, I83.029, I83.10, I83.11, I83.20, I83.202, I83.203, I83.205, I83.208, I83.209, I83.211, I83.212, I83.218, I83.22, I83.221, I83.224, I83.228, I83.229, I83.8, I83.81, I83.813, I83.89, I83.893, I83.91, I83.92, I83.93, I85, I85.01, I85.1, I85.10, I86.0, I86.3, I86.4, I86.8, I87, I87.0, I87.001, I87.003, I87.012, I87.013, I87.019, I87.021, I87.022, I87.023, I87.029, I87.032, I87.033, I87.039, I87.093, I87.099, I87.2, I87.3, I87.302, I87.313, I87.329, I87.333, I87.39, I87.391, I87.399, I87.9, I88, I88.9, I89.0, I89.8, I89.9, I95, I95.0, I95.1, I95.8, I95.81, I95.9, I97, I97.0, I97.11, I97.110, I97.111, I97.12, I97.121, I97.13, I97.130, I97.131, I97.190, I97.2, I97.4, I97.41, I97.410, I97.418, I97.5, I97.51, I97.6, I97.61, I97.611, I97.63, I97.630, I97.64, I97.640, I97.71, I97.710, I97.711, I97.791, I97.8, I97.811, I97.820, I97.88, I99.8, I99.9 |
| Chronic Neurological Disorders | Any medical claim, inpatient hospital encounter, or outpatient hospital encounter with one of the following ICD-10-CM codes: G10, G11, G11.0, G11.1, G11.2, G11.3, G11.4, G11.8, G11.9, G12, G12.0, G12.1, G12.2, G12.20, G12.21, G12.22, G12.23, G12.24, G12.25, G12.29, G12.8, G12.9, G13, G13.0, G13.1, G13.2, G13.8, G20, G21, G21.0, G21.1, G21.11, G21.19, G21.2, G21.3, G21.4, G21.8, G21.9, G25.4, G25.5, G31.2, G31.8, G31.81, G31.82, G31.83, G31.84, G31.85, G31.89, G31.9, G32, G32.0, G32.8, G32.81, G32.89, G35, G36, G36.0, G36.1, G36.8, G36.9, G37, G37.0, G37.1, G37.2, G37.3, G37.4, G37.5, G37.8, G37.9, G40, G40.0, G40.00, G40.001, G40.009, G40.01, G40.011, G40.019, G40.1, G40.10, G40.101, G40.109, G40.11, G40.111, G40.119, G40.2, G40.20, G40.201, G40.209, G40.21, G40.211, G40.219, G40.3, G40.30, G40.301, G40.309, G40.31, G40.311, G40.319, G40.4, G40.40, G40.401, G40.409, G40.41, G40.411, G40.419, G40.5, G40.50, G40.501, G40.509, G40.8, G40.80, G40.801, G40.802, G40.803, G40.804, G40.81, G40.811, G40.812, G40.813, G40.814, G40.82, G40.821, G40.822, G40.823, G40.824, G40.89, G40.9, G40.90, G40.901, G40.909, G40.91, G40.911, G40.919, G40.A, G40.A0, G40.A01, G40.A09, G40.A1, G40.A11, G40.A19, G40.B, G40.B0, G40.B01, G40.B09, G40.B1, G40.B11, G40.B19, G93.1, G93.4, G93.40, G93.41, G93.49, R47.0, R47.01, R47.02, R56, R56.0, R56.00, R56.01, R56.1, R56.9 |
| Immunosuppression | Any medical claim, inpatient hospital encounter, or outpatient hospital encounter with one of the following ICD-10-CM codes: C00.1, C00.4, C01, C02.2, C02.4, C02.8, C02.9, C03, C03.0, C04.0, C04.8, C04.9, C05.1, C05.2, C05.9, C06, C06.0, C06.2, C06.80, C06.89, C11, C11.1, C11.2, C12, C13.0, C13.1, C13.2, C13.9, C14.2, C15.5, C15.8, C16, C16.0, C16.1, C16.2, C16.3, C16.4, C16.9, C17, C17.0, C17.8, C17.9, C18, C18.3, C18.7, C18.8, C19, C22, C22.2, C22.4, C22.8, C22.9, C23, C24.0, C24.8, C24.9, C25, C25.2, C25.3, C25.4, C26.0, C26.1, C26.9, C30, C30.0, C30.1, C31, C31.0, C31.1, C31.2, C31.3, C31.8, C31.9, C32, C32.0, C32.3, C32.9, C33, C34.0, C34.1, C34.2, C34.31, C34.8, C34.82, C34.9, C34.90, C34.92, C38.0, C38.2, C38.3, C38.4, C39.9, C40, C40.10, C40.11, C40.2, C40.20, C40.3, C40.30, C40.31, C40.8, C40.80, C41, C41.4, C41.9, C43.1, C43.112, C43.121, C43.20, C43.22, C43.30, C43.31, C43.5, C43.52, C43.6, C43.61, C43.62, C43.70, C43.71, C43.9, C44.09, C44.10, C44.101, C44.1022, C44.109, C44.1091, C44.111, C44.112, C44.1221, C44.129, C44.1291, C44.13, C44.131, C44.132, C44.1321, C44.1322, C44.139, C44.1392, C44.19, C44.191, C44.1921, C44.1922, C44.202, C44.209, C44.219, C44.221, C44.222, C44.229, C44.292, C44.299, C44.3, C44.30, C44.300, C44.31, C44.319, C44.320, C44.39, C44.390, C44.391, C44.399, C44.4, C44.40, C44.49, C44.500, C44.510, C44.511, C44.519, C44.52, C44.520, C44.521, C44.59, C44.590, C44.599, C44.602, C44.62, C44.621, C44.629, C44.692, C44.70, C44.702, C44.709, C44.71, C44.711, C44.712, C44.719, C44.721, C44.722, C44.79, C44.8, C44.80, C44.89, C44.90, C45.1, C45.2, C45.7, C45.9, C46.0, C46.1, C46.3, C46.4, C46.51, C47, C47.10, C47.11, C47.20, C47.21, C47.22, C47.3, C47.4, C47.5, C47.9, C48, C48.0, C48.2, C48.8, C49.0, C49.20, C49.22, C49.5, C49.8, C49.9, C49.A0, C49.A2, C49.A5, C50.0, C50.019, C50.02, C50.1, C50.11, C50.112, C50.121, C50.122, C50.129, C50.2, C50.211, C50.212, C50.221, C50.312, C50.32, C50.321, C50.329, C50.4, C50.412, C50.419, C50.421, C50.422, C50.5, C50.512, C50.519, C50.52, C50.521, C50.522, C50.6, C50.61, C50.611, C50.619, C50.629, C50.811, C50.812, C50.82, C50.822, C50.9, C50.911, C50.912, C50.919, C50.922, C51, C51.0, C51.1, C51.8, C51.9, C53.8, C53.9, C54.2, C54.3, C54.8, C54.9, C56, C56.1, C57, C57.0, C57.00, C57.01, C57.02, C57.10, C57.2, C57.20, C57.21, C57.22, C57.3, C57.4, C57.9, C58, C60.2, C60.9, C62.0, C62.02, C62.91, C63, C63.0, C63.01, C63.10, C63.11, C63.12, C63.7, C64, C64.1, C64.2, C66.1, C66.2, C66.9, C67, C67.1, C67.2, C67.6, C67.8, C67.9, C68.0, C68.8, C69.00, C69.02, C69.12, C69.21, C69.22, C69.30, C69.4, C69.41, C69.50, C69.52, C69.60, C69.61, C69.8, C69.80, C69.9, C69.90, C70.0, C70.9, C71.0, C71.3, C71.4, C71.5, C71.6, C71.8, C71.9, C72, C72.1, C72.2, C72.21, C72.3, C72.40, C72.42, C72.59, C72.9, C73, C74.0, C74.01, C74.02, C74.11, C74.12, C74.9, C74.90, C75, C75.3, C75.5, C75.9, C76.0, C76.1, C76.4, C76.5, C76.50, C76.51, C76.52, C77.0, C77.2, C77.3, C77.4, C77.9, C78.0, C78.00, C78.01, C78.02, C78.1, C78.30, C78.39, C78.7, C78.80, C79.1, C79.32, C79.4, C79.5, C79.51, C79.52, C79.60, C79.62, C79.7, C79.70, C79.72, C79.8, C79.81, C79.82, C79.89, C79.9, C7A, C80, C80.0, C80.1, C80.2, C81, C81.0, C81.00, C81.05, C81.06, C81.08, C81.12, C81.14, C81.17, C81.19, C81.2, C81.21, C81.27, C81.31, C81.33, C81.35, C81.36, C81.37, C81.4, C81.41, C81.42, C81.44, C81.45, C81.46, C81.47, C81.48, C81.49, C81.7, C81.71, C81.72, C81.73, C81.74, C81.76, C81.77, C81.90, C81.95, C81.96, C81.97, C81.99, C82.0, C82.00, C82.01, C82.02, C82.03, C82.09, C82.1, C82.10, C82.11, C82.13, C82.14, C82.17, C82.18, C82.19, C82.20, C82.23, C82.29, C82.30, C82.34, C82.35, C82.36, C82.38, C82.39, C82.43, C82.45, C82.49, C82.51, C82.52, C82.53, C82.54, C82.55, C82.56, C82.57, C82.60, C82.65, C82.66, C82.67, C82.68, C82.83, C82.84, C82.85, C82.86, C82.89, C82.93, C82.95, C82.97, C82.99, C83.03, C83.04, C83.06, C83.08, C83.1, C83.11, C83.12, C83.13, C83.15, C83.19, C83.32, C83.33, C83.35, C83.50, C83.51, C83.52, C83.54, C83.55, C83.56, C83.57, C83.58, C83.7, C83.70, C83.71, C83.72, C83.74, C83.75, C83.78, C83.79, C83.8, C83.81, C83.84, C83.85, C83.87, C83.88, C83.89, C83.91, C83.92, C83.93, C83.98, C83.99, C88, C88.2, C88.3, C90.10, C90.12, C90.3, C90.31, C90.32, C91.0, C91.01, C91.1, C91.10, C91.12, C91.3, C91.40, C91.41, C91.50, C91.52, C91.6, C91.60, C91.61, C91.91, C91.92, C91.A, C91.A1, C91.Z1, C92, C92.01, C92.02, C92.1, C92.11, C92.12, C92.2, C92.20, C92.3, C92.30, C92.31, C92.41, C92.42, C92.50, C92.60, C92.9, C92.A, C92.A2, C92.Z0, C92.Z1, C92.Z2, C93.0, C93.10, C93.3, C93.30, C93.32, C93.9, C93.90, C93.92, C94, C94.02, C94.2, C94.20, C94.21, C94.22, C94.4, C94.8, C95.00, C95.01, C95.1, C95.10, C95.11, C95.12, C95.90, C95.91, C95.92, C96, C96.0, C96.2, C96.22, C96.5, C96.6, C96.A, C96.Z, D00.00, D00.01, D00.02, D00.04, D00.05, D00.06, D00.07, D00.08, D01.0, D01.40, D01.5, D01.9, D02, D02.1, D02.20, D02.21, D03.0, D03.11, D03.12, D03.20, D03.22, D03.30, D03.4, D03.5, D03.6, D03.61, D03.7, D03.71, D04.0, D04.1, D04.12, D04.2, D04.20, D04.21, D04.30, D04.39, D04.4, D04.5, D04.6, D04.62, D04.7, D04.70, D04.71, D04.72, D04.8, D04.9, D05, D05.00, D05.01, D05.1, D05.10, D05.12, D05.8, D05.80, D05.81, D05.9, D05.90, D05.91, D05.92, D06.0, D06.7, D07, D07.0, D07.2, D07.39, D07.4, D07.6, D07.60, D07.61, D07.69, D09, D09.2, D09.21, D09.22, D09.9, D10.1, D10.30, D10.39, D10.4, D10.5, D10.6, D10.7, D11.0, D11.7, D11.9, D12, D12.2, D12.3, D12.5, D12.6, D12.7, D13, D13.1, D13.3, D13.30, D13.5, D13.6, D14.0, D14.1, D15.9, D16, D16.00, D16.01, D16.1, D16.11, D16.12, D16.20, D16.21, D16.22, D16.3, D16.5, D16.6, D16.9, D17.0, D17.2, D17.3, D17.39, D17.6, D17.72, D18.00, D18.09, D18.1, D19, D19.0, D20, D20.0, D20.1, D21, D21.10, D21.11, D21.20, D21.21, D21.3, D21.5, D22, D22.1, D22.10, D22.111, D22.112, D22.12, D22.122, D22.2, D22.20, D22.22, D22.4, D22.5, D22.6, D22.61, D22.62, D22.72, D22.9, D23, D23.0, D23.1, D23.11, D23.111, D23.112, D23.12, D23.122, D23.20, D23.21, D23.22, D23.39, D23.5, D23.61, D23.70, D23.71, D23.72, D23.9, D24, D24.2, D25.2, D26, D26.0, D26.1, D26.9, D27.1, D28, D28.0, D28.7, D29, D29.0, D29.3, D29.30, D29.31, D29.32, D29.4, D29.8, D30.00, D30.02, D30.1, D30.2, D30.21, D30.22, D30.3, D30.4, D31.01, D31.10, D31.11, D31.3, D31.42, D31.5, D31.6, D31.90, D32, D32.9, D33.0, D33.2, D33.4, D33.9, D34, D35.2, D35.5, D35.9, D36, D36.0, D36.1, D36.10, D36.11, D36.12, D36.13, D36.15, D36.9, D37.01, D37.02, D37.05, D37.3, D37.6, D37.8, D38, D38.0, D38.1, D38.4, D38.5, D38.6, D39.1, D39.11, D39.12, D39.2, D40, D40.10, D41, D41.01, D41.1, D41.11, D41.8, D41.9, D42, D42.1, D43.0, D43.1, D44, D44.11, D44.4, D44.6, D44.9, D45, D46, D46.20, D46.4, D46.9, D46.A, D46.C, D47.4, D47.9, D47.Z1, D47.Z9, D48.2, D48.4, D48.5, D48.60, D48.61, D49, D49.0, D49.1, D49.3, D49.4, D49.51, D49.511, D49.519, D49.6, D49.7, D49.8, D49.9, D70.3, D70.8, D70.9, D72.0, D72.810, D72.819, D75.81, D75.89, D75.9, D86, D89.0, D89.2, D89.40, D89.41, D89.43, D89.810, D89.811, D89.812, D89.813, D89.82, D89.9, E85.8, G35, G37.1, G37.8, G61.9, I40, I82.C12, J84.02, K50.013, K50.014, K50.018, K50.019, K50.10, K50.112, K50.114, K50.118, K50.119, K50.8, K50.81, K50.818, K50.819, K50.9, K50.90, K50.911, K50.912, K50.914, K50.918, K51.011, K51.013, K51.018, K51.019, K51.2, K51.20, K51.21, K51.213, K51.214, K51.219, K51.3, K51.31, K51.313, K51.314, K51.318, K51.412, K51.413, K51.414, K51.419, K51.5, K51.51, K51.512, K51.8, K51.80, K51.81, K51.811, K51.818, K51.819, K51.91, K51.913, K52, K52.0, K52.1, K52.2, K52.22, K52.29, K52.3, K52.81, K52.831, K52.832, K52.838, K52.839, K52.89, L93.0, M05, M05.0, M05.011, M05.012, M05.02, M05.029, M05.03, M05.032, M05.04, M05.041, M05.049, M05.052, M05.059, M05.062, M05.069, M05.09, M05.10, M05.11, M05.111, M05.119, M05.12, M05.129, M05.131, M05.132, M05.139, M05.149, M05.15, M05.159, M05.169, M05.179, M05.2, M05.20, M05.211, M05.212, M05.221, M05.239, M05.24, M05.249, M05.252, M05.259, M05.261, M05.262, M05.269, M05.27, M05.272, M05.279, M05.3, M05.31, M05.312, M05.319, M05.32, M05.321, M05.322, M05.332, M05.339, M05.34, M05.35, M05.351, M05.359, M05.36, M05.361, M05.369, M05.371, M05.379, M05.39, M05.411, M05.421, M05.422, M05.429, M05.43, M05.431, M05.432, M05.451, M05.459, M05.462, M05.49, M05.511, M05.521, M05.532, M05.539, M05.54, M05.542, M05.552, M05.559, M05.56, M05.569, M05.571, M05.572, M05.59, M05.61, M05.619, M05.622, M05.629, M05.639, M05.64, M05.642, M05.649, M05.65, M05.651, M05.659, M05.66, M05.662, M05.669, M05.672, M05.7, M05.71, M05.711, M05.722, M05.73, M05.739, M05.74, M05.749, M05.75, M05.759, M05.761, M05.762, M05.769, M05.771, M05.772, M05.779, M05.79, M05.8, M05.811, M05.819, M05.821, M05.822, M05.83, M05.839, M05.841, M05.842, M05.849, M05.859, M05.86, M05.861, M05.862, M05.869, M05.871, M05.872, M05.89, M06.0, M06.00, M06.011, M06.029, M06.031, M06.032, M06.039, M06.04, M06.041, M06.042, M06.049, M06.051, M06.06, M06.062, M06.07, M06.071, M06.072, M06.212, M06.22, M06.221, M06.222, M06.24, M06.242, M06.251, M06.252, M06.262, M06.269, M06.272, M06.28, M06.29, M06.3, M06.30, M06.311, M06.32, M06.321, M06.322, M06.329, M06.331, M06.332, M06.339, M06.361, M06.362, M06.369, M06.37, M06.371, M06.372, M06.379, M06.38, M06.812, M06.82, M06.821, M06.831, M06.832, M06.839, M06.851, M06.852, M06.86, M06.861, M06.871, M06.872, M06.879, M06.89, M07.60, M07.61, M07.611, M07.631, M07.64, M07.642, M07.65, M07.66, M07.661, M07.662, M07.669, M07.671, M07.672, M07.68, M08, M08.00, M08.01, M08.011, M08.019, M08.02, M08.022, M08.031, M08.039, M08.041, M08.052, M08.059, M08.06, M08.062, M08.07, M08.08, M08.1, M08.211, M08.221, M08.252, M08.272, M08.28, M08.29, M08.3, M08.4, M08.411, M08.412, M08.42, M08.421, M08.429, M08.431, M08.44, M08.442, M08.449, M08.47, M08.471, M08.80, M08.811, M08.82, M08.822, M08.829, M08.83, M08.832, M08.85, M08.851, M08.86, M08.861, M08.862, M08.869, M08.87, M08.871, M08.872, M08.879, M08.88, M08.89, M08.9, M08.91, M08.919, M08.92, M08.93, M08.931, M08.932, M08.941, M08.942, M08.949, M08.951, M08.952, M08.96, M08.969, M08.979, M08.99, M10.0, M10.01, M10.011, M10.012, M10.021, M10.029, M10.03, M10.031, M10.032, M10.039, M10.041, M10.042, M10.05, M10.051, M10.06, M10.061, M10.062, M10.07, M10.072, M10.08, M10.1, M10.10, M10.111, M10.119, M10.12, M10.121, M10.122, M10.129, M10.131, M10.141, M10.15, M10.152, M10.159, M10.16, M10.179, M10.18, M10.19, M10.20, M10.211, M10.212, M10.219, M10.221, M10.229, M10.23, M10.24, M10.241, M10.249, M10.25, M10.251, M10.252, M10.26, M10.262, M10.269, M10.279, M10.28, M10.29, M10.30, M10.31, M10.311, M10.312, M10.32, M10.34, M10.341, M10.35, M10.36, M10.361, M10.369, M10.37, M10.372, M10.38, M10.40, M10.41, M10.411, M10.419, M10.421, M10.422, M10.43, M10.432, M10.44, M10.441, M10.442, M10.45, M10.452, M10.46, M10.461, M10.47, M10.471, M10.472, M10.48, M11, M11.00, M11.01, M11.011, M11.019, M11.02, M11.021, M11.029, M11.032, M11.039, M11.04, M11.042, M11.049, M11.05, M11.052, M11.06, M11.1, M11.10, M11.112, M11.119, M11.129, M11.13, M11.15, M11.151, M11.152, M11.161, M11.162, M11.17, M11.171, M11.179, M11.19, M11.2, M11.20, M11.21, M11.212, M11.219, M11.229, M11.23, M11.231, M11.239, M11.241, M11.242, M11.251, M11.259, M11.26, M11.261, M11.271, M11.28, M11.29, M11.8, M11.80, M11.81, M11.819, M11.82, M11.821, M11.83, M11.84, M11.841, M11.842, M11.859, M11.861, M11.88, M11.89, M12.0, M12.01, M12.019, M12.021, M12.029, M12.039, M12.04, M12.041, M12.042, M12.049, M12.05, M12.051, M12.052, M12.059, M12.06, M12.061, M12.069, M12.071, M12.072, M12.079, M12.08, M12.09, M12.10, M12.12, M12.122, M12.129, M12.13, M12.131, M12.132, M12.139, M12.15, M12.152, M12.16, M12.172, M12.20, M12.21, M12.211, M12.212, M12.22, M12.229, M12.23, M12.231, M12.232, M12.239, M12.24, M12.241, M12.249, M12.251, M12.252, M12.262, M12.279, M12.30, M12.312, M12.319, M12.322, M12.329, M12.331, M12.332, M12.339, M12.34, M12.35, M12.359, M12.36, M12.362, M12.369, M12.372, M12.38, M12.39, M12.4, M12.411, M12.419, M12.421, M12.43, M12.431, M12.449, M12.45, M12.451, M12.452, M12.459, M12.46, M12.461, M12.462, M12.47, M12.471, M12.479, M12.48, M12.5, M12.51, M12.511, M12.52, M12.522, M12.531, M12.539, M12.54, M12.542, M12.549, M12.55, M12.551, M12.552, M12.569, M12.57, M12.571, M12.59, M12.8, M12.81, M12.812, M12.819, M12.821, M12.822, M12.839, M12.842, M12.851, M12.862, M12.869, M12.871, M12.872, M12.879, M12.88, M13.0, M13.10, M13.11, M13.111, M13.13, M13.139, M13.141, M13.142, M13.152, M13.161, M13.162, M13.17, M13.171, M13.179, M13.8, M13.80, M13.811, M13.812, M13.819, M13.82, M13.821, M13.822, M13.829, M13.83, M13.831, M13.832, M13.839, M13.841, M13.851, M13.862, M13.869, M13.87, M14, M14.60, M14.611, M14.612, M14.629, M14.632, M14.639, M14.64, M14.649, M14.659, M14.661, M14.67, M14.671, M14.679, M14.68, M14.69, M14.80, M14.81, M14.811, M14.819, M14.82, M14.829, M14.831, M14.841, M14.85, M14.851, M14.852, M14.859, M14.862, M14.879, M14.88, M31.5, M32, M35.3, M35.8, M35.9, R76, R76.0, R76.12, R76.8, R83.4, R83.5, R83.6, R83.9, R84, R84.0, R84.6, R84.8, R85.3, R85.6, R85.61, R85.612, R85.618, R85.8, R85.81, R85.82, R85.89, R85.9, R86, R86.0, R86.1, R86.2, R86.3, R86.5, R86.7, R86.9, R87, R87.2, R87.3, R87.4, T78.40, Z94, B20, C00, C00.0, C00.2, C00.3, C00.5, C00.6, C00.8, C00.9, C02, C02.0, C02.1, C02.3, C03.1, C03.9, C04, C04.1, C05, C05.0, C05.8, C06.1, C06.8, C06.9, C07, C11.0, C11.3, C11.8, C11.9, C13, C13.8, C14, C14.0, C14.8, C15, C15.3, C15.4, C15.9, C16.5, C16.6, C16.8, C17.1, C17.2, C17.3, C18.0, C18.1, C18.2, C18.4, C18.5, C18.6, C18.9, C22.0, C22.1, C22.3, C22.7, C24, C24.1, C25.0, C25.1, C25.7, C25.8, C25.9, C26, C32.1, C32.2, C32.8, C34, C34.00, C34.01, C34.02, C34.10, C34.11, C34.12, C34.3, C34.30, C34.32, C34.80, C34.81, C34.91, C37, C38, C38.1, C38.8, C39, C39.0, C40.0, C40.00, C40.01, C40.02, C40.1, C40.12, C40.21, C40.22, C40.32, C40.81, C40.82, C40.9, C40.90, C40.91, C40.92, C41.0, C41.1, C41.2, C41.3, C43, C43.0, C43.10, C43.11, C43.111, C43.12, C43.122, C43.2, C43.21, C43.3, C43.39, C43.4, C43.51, C43.59, C43.60, C43.7, C43.72, C43.8, C44, C44.0, C44.00, C44.01, C44.02, C44.1, C44.102, C44.1021, C44.1092, C44.11, C44.1121, C44.1122, C44.119, C44.1191, C44.1192, C44.12, C44.121, C44.122, C44.1222, C44.1292, C44.1391, C44.192, C44.199, C44.1991, C44.1992, C44.2, C44.20, C44.201, C44.21, C44.211, C44.212, C44.22, C44.29, C44.291, C44.301, C44.309, C44.310, C44.311, C44.32, C44.321, C44.329, C44.41, C44.42, C44.5, C44.50, C44.501, C44.509, C44.51, C44.529, C44.591, C44.6, C44.60, C44.601, C44.609, C44.61, C44.611, C44.612, C44.619, C44.622, C44.69, C44.691, C44.699, C44.7, C44.701, C44.72, C44.729, C44.791, C44.792, C44.799, C44.81, C44.82, C44.9, C44.91, C44.92, C44.99, C45, C45.0, C46, C46.2, C46.5, C46.50, C46.52, C46.7, C46.9, C47.0, C47.1, C47.12, C47.2, C47.6, C47.8, C48.1, C49, C49.1, C49.10, C49.11, C49.12, C49.2, C49.21, C49.3, C49.4, C49.6, C49.A, C49.A1, C49.A3, C49.A4, C49.A9, C50, C50.01, C50.011, C50.012, C50.021, C50.022, C50.029, C50.111, C50.119, C50.12, C50.21, C50.219, C50.22, C50.222, C50.229, C50.3, C50.31, C50.311, C50.319, C50.322, C50.41, C50.411, C50.42, C50.429, C50.51, C50.511, C50.529, C50.612, C50.62, C50.621, C50.622, C50.8, C50.81, C50.819, C50.821, C50.829, C50.91, C50.92, C50.921, C50.929, C51.2, C52, C53, C53.0, C53.1, C54, C54.0, C54.1, C55, C56.2, C56.9, C57.1, C57.11, C57.12, C57.7, C57.8, C60, C60.0, C60.1, C60.8, C61, C62, C62.00, C62.01, C62.1, C62.10, C62.11, C62.12, C62.9, C62.90, C62.92, C63.00, C63.02, C63.1, C63.2, C63.8, C63.9, C64.9, C65, C65.1, C65.2, C65.9, C66, C67.0, C67.3, C67.4, C67.5, C67.7, C68, C68.1, C68.9, C69, C69.0, C69.01, C69.1, C69.10, C69.11, C69.2, C69.20, C69.3, C69.31, C69.32, C69.40, C69.42, C69.5, C69.51, C69.6, C69.62, C69.81, C69.82, C69.91, C69.92, C70, C70.1, C71, C71.1, C71.2, C71.7, C72.0, C72.20, C72.22, C72.30, C72.31, C72.32, C72.4, C72.41, C72.5, C72.50, C74, C74.00, C74.1, C74.10, C74.91, C74.92, C75.0, C75.1, C75.2, C75.4, C75.8, C76, C76.2, C76.3, C76.40, C76.41, C76.42, C76.8, C77, C77.1, C77.5, C77.8, C78, C78.2, C78.3, C78.4, C78.5, C78.6, C78.8, C78.89, C79, C79.0, C79.00, C79.01, C79.02, C79.10, C79.11, C79.19, C79.2, C79.3, C79.31, C79.40, C79.49, C79.6, C79.61, C79.71, C7B, C81.01, C81.02, C81.03, C81.04, C81.07, C81.09, C81.1, C81.10, C81.11, C81.13, C81.15, C81.16, C81.18, C81.20, C81.22, C81.23, C81.24, C81.25, C81.26, C81.28, C81.29, C81.3, C81.30, C81.32, C81.34, C81.38, C81.39, C81.40, C81.43, C81.70, C81.75, C81.78, C81.79, C81.9, C81.91, C81.92, C81.93, C81.94, C81.98, C82, C82.04, C82.05, C82.06, C82.07, C82.08, C82.12, C82.15, C82.16, C82.2, C82.21, C82.22, C82.24, C82.25, C82.26, C82.27, C82.28, C82.3, C82.31, C82.32, C82.33, C82.37, C82.4, C82.40, C82.41, C82.42, C82.44, C82.46, C82.47, C82.48, C82.5, C82.50, C82.58, C82.59, C82.6, C82.61, C82.62, C82.63, C82.64, C82.69, C82.8, C82.80, C82.81, C82.82, C82.87, C82.88, C82.9, C82.90, C82.91, C82.92, C82.94, C82.96, C82.98, C83, C83.0, C83.00, C83.01, C83.02, C83.05, C83.07, C83.09, C83.10, C83.14, C83.16, C83.17, C83.18, C83.3, C83.30, C83.31, C83.34, C83.36, C83.37, C83.38, C83.39, C83.5, C83.53, C83.59, C83.73, C83.76, C83.77, C83.80, C83.82, C83.83, C83.86, C83.9, C83.90, C83.94, C83.95, C83.96, C83.97, C88.0, C88.4, C88.8, C88.9, C90, C90.0, C90.00, C90.01, C90.02, C90.1, C90.11, C90.2, C90.20, C90.21, C90.22, C90.30, C91, C91.00, C91.02, C91.11, C91.30, C91.31, C91.32, C91.4, C91.42, C91.5, C91.51, C91.62, C91.9, C91.90, C91.A0, C91.A2, C91.Z, C91.Z0, C91.Z2, C92.0, C92.00, C92.10, C92.21, C92.22, C92.32, C92.4, C92.40, C92.5, C92.51, C92.52, C92.6, C92.61, C92.62, C92.90, C92.91, C92.92, C92.A0, C92.A1, C92.Z, C93, C93.00, C93.01, C93.02, C93.1, C93.11, C93.12, C93.31, C93.91, C93.Z, C93.Z0, C93.Z1, C93.Z2, C94.0, C94.00, C94.01, C94.3, C94.30, C94.31, C94.32, C94.40, C94.41, C94.42, C94.6, C94.80, C94.81, C94.82, C95, C95.0, C95.02, C95.9, C96.20, C96.21, C96.29, C96.4, C96.9, D00, D00.0, D00.03, D00.1, D00.2, D01, D01.1, D01.2, D01.3, D01.4, D01.49, D01.7, D02.0, D02.2, D02.22, D02.3, D02.4, D03, D03.1, D03.10, D03.111, D03.112, D03.121, D03.122, D03.2, D03.21, D03.3, D03.39, D03.51, D03.52, D03.59, D03.60, D03.62, D03.70, D03.72, D03.8, D03.9, D04, D04.10, D04.11, D04.111, D04.112, D04.121, D04.122, D04.22, D04.3, D04.60, D04.61, D05.0, D05.02, D05.11, D05.82, D06, D06.1, D06.9, D07.1, D07.3, D07.30, D07.5, D09.0, D09.1, D09.10, D09.19, D09.20, D09.3, D09.8, D10, D10.0, D10.2, D10.3, D10.9, D11, D12.0, D12.1, D12.4, D12.8, D12.9, D13.0, D13.2, D13.39, D13.4, D13.7, D13.9, D14, D14.2, D14.3, D14.30, D14.31, D14.32, D14.4, D15, D15.0, D15.1, D15.2, D15.7, D16.0, D16.02, D16.10, D16.2, D16.30, D16.31, D16.32, D16.4, D16.7, D16.8, D17, D17.1, D17.20, D17.21, D17.22, D17.23, D17.24, D17.30, D17.4, D17.5, D17.7, D17.71, D17.79, D17.9, D18, D18.0, D18.01, D18.02, D18.03, D19.1, D19.7, D19.9, D21.0, D21.1, D21.12, D21.2, D21.22, D21.4, D21.6, D21.9, D22.0, D22.11, D22.121, D22.21, D22.3, D22.30, D22.39, D22.60, D22.7, D22.70, D22.71, D23.10, D23.121, D23.2, D23.3, D23.30, D23.4, D23.6, D23.60, D23.62, D23.7, D24.1, D24.9, D25, D25.0, D25.1, D25.9, D26.7, D27, D27.0, D27.9, D28.1, D28.2, D28.9, D29.1, D29.2, D29.20, D29.21, D29.22, D29.9, D30, D30.0, D30.01, D30.10, D30.11, D30.12, D30.20, D30.8, D30.9, D31, D31.0, D31.00, D31.02, D31.1, D31.12, D31.2, D31.20, D31.21, D31.22, D31.30, D31.31, D31.32, D31.4, D31.40, D31.41, D31.50, D31.51, D31.52, D31.60, D31.61, D31.62, D31.9, D31.91, D31.92, D32.0, D32.1, D33, D33.1, D33.3, D33.7, D35, D35.0, D35.00, D35.01, D35.02, D35.1, D35.3, D35.4, D35.6, D35.7, D36.14, D36.16, D36.17, D36.7, D37, D37.0, D37.03, D37.030, D37.031, D37.032, D37.039, D37.04, D37.09, D37.1, D37.2, D37.4, D37.5, D37.9, D38.2, D38.3, D39, D39.0, D39.10, D39.8, D39.9, D3A, D40.0, D40.1, D40.11, D40.12, D40.8, D40.9, D41.0, D41.00, D41.02, D41.10, D41.12, D41.2, D41.20, D41.21, D41.22, D41.3, D41.4, D42.0, D42.9, D43, D43.2, D43.3, D43.4, D43.8, D43.9, D44.0, D44.1, D44.10, D44.12, D44.2, D44.3, D44.5, D44.7, D46.0, D46.1, D46.2, D46.21, D46.22, D46.B, D46.Z, D47, D47.0, D47.01, D47.02, D47.09, D47.1, D47.2, D47.3, D47.Z, D47.Z2, D48, D48.0, D48.1, D48.3, D48.6, D48.62, D48.7, D48.9, D49.2, D49.5, D49.512, D49.59, D49.81, D49.89, D70, D70.0, D70.1, D70.2, D70.4, D71, D72.81, D72.818, D72.9, D89, D89.1, D89.3, D89.4, D89.42, D89.49, D89.8, D89.81, D89.89, E85, E85.0, E85.1, E85.3, G36, G37.3, G37.9, G61.0, I82.C11, J67.9, J84.01, J84.09, K50, K50.0, K50.00, K50.01, K50.011, K50.012, K50.1, K50.11, K50.111, K50.113, K50.80, K50.811, K50.812, K50.813, K50.814, K50.91, K50.913, K50.919, K51, K51.0, K51.00, K51.01, K51.012, K51.014, K51.211, K51.212, K51.218, K51.30, K51.311, K51.312, K51.319, K51.4, K51.40, K51.41, K51.411, K51.418, K51.50, K51.511, K51.513, K51.514, K51.518, K51.519, K51.812, K51.813, K51.814, K51.9, K51.90, K51.911, K51.912, K51.914, K51.918, K51.919, K52.21, K52.8, K52.82, K52.83, K52.9, L93.2, L94, M01.X0, M02.10, M04, M05.00, M05.01, M05.019, M05.021, M05.022, M05.031, M05.039, M05.042, M05.05, M05.051, M05.06, M05.061, M05.07, M05.071, M05.072, M05.079, M05.1, M05.112, M05.121, M05.122, M05.13, M05.14, M05.141, M05.142, M05.151, M05.152, M05.16, M05.161, M05.162, M05.17, M05.171, M05.172, M05.19, M05.21, M05.219, M05.22, M05.222, M05.229, M05.23, M05.231, M05.232, M05.241, M05.242, M05.25, M05.251, M05.26, M05.271, M05.29, M05.30, M05.311, M05.329, M05.33, M05.331, M05.341, M05.342, M05.349, M05.352, M05.362, M05.37, M05.372, M05.4, M05.40, M05.41, M05.412, M05.419, M05.42, M05.439, M05.44, M05.441, M05.442, M05.449, M05.45, M05.452, M05.46, M05.461, M05.469, M05.47, M05.471, M05.472, M05.479, M05.5, M05.50, M05.51, M05.512, M05.519, M05.52, M05.522, M05.529, M05.53, M05.531, M05.541, M05.549, M05.55, M05.551, M05.561, M05.562, M05.57, M05.579, M05.6, M05.60, M05.611, M05.612, M05.62, M05.621, M05.63, M05.631, M05.632, M05.641, M05.652, M05.661, M05.67, M05.671, M05.679, M05.69, M05.70, M05.712, M05.719, M05.72, M05.721, M05.729, M05.731, M05.732, M05.741, M05.742, M05.751, M05.752, M05.76, M05.77, M05.80, M05.81, M05.812, M05.82, M05.829, M05.831, M05.832, M05.84, M05.85, M05.851, M05.852, M05.87, M05.879, M05.9, M06, M06.01, M06.012, M06.019, M06.02, M06.021, M06.022, M06.03, M06.05, M06.052, M06.059, M06.061, M06.069, M06.079, M06.08, M06.09, M06.1, M06.2, M06.20, M06.21, M06.211, M06.219, M06.229, M06.23, M06.231, M06.232, M06.239, M06.241, M06.249, M06.25, M06.259, M06.26, M06.261, M06.27, M06.271, M06.279, M06.31, M06.312, M06.319, M06.33, M06.34, M06.341, M06.342, M06.349, M06.35, M06.351, M06.352, M06.359, M06.36, M06.39, M06.4, M06.8, M06.80, M06.81, M06.811, M06.819, M06.822, M06.829, M06.83, M06.84, M06.841, M06.842, M06.849, M06.85, M06.859, M06.862, M06.869, M06.87, M06.88, M06.9, M07, M07.6, M07.612, M07.619, M07.62, M07.621, M07.622, M07.629, M07.63, M07.632, M07.639, M07.641, M07.649, M07.651, M07.652, M07.659, M07.67, M07.679, M07.69, M08.0, M08.012, M08.021, M08.029, M08.03, M08.032, M08.04, M08.042, M08.049, M08.05, M08.051, M08.061, M08.069, M08.071, M08.072, M08.079, M08.09, M08.2, M08.20, M08.21, M08.212, M08.219, M08.22, M08.222, M08.229, M08.23, M08.231, M08.232, M08.239, M08.24, M08.241, M08.242, M08.249, M08.25, M08.251, M08.259, M08.26, M08.261, M08.262, M08.269, M08.27, M08.271, M08.279, M08.40, M08.41, M08.419, M08.422, M08.43, M08.432, M08.439, M08.441, M08.45, M08.451, M08.452, M08.459, M08.46, M08.461, M08.462, M08.469, M08.472, M08.479, M08.48, M08.8, M08.81, M08.812, M08.819, M08.821, M08.831, M08.839, M08.84, M08.841, M08.842, M08.849, M08.852, M08.859, M08.90, M08.911, M08.912, M08.921, M08.922, M08.929, M08.939, M08.94, M08.95, M08.959, M08.961, M08.962, M08.97, M08.971, M08.972, M08.98, M10, M10.00, M10.019, M10.02, M10.022, M10.04, M10.049, M10.052, M10.059, M10.069, M10.071, M10.079, M10.09, M10.11, M10.112, M10.13, M10.132, M10.139, M10.14, M10.142, M10.149, M10.151, M10.161, M10.162, M10.169, M10.17, M10.171, M10.172, M10.2, M10.21, M10.22, M10.222, M10.231, M10.232, M10.239, M10.242, M10.259, M10.261, M10.27, M10.271, M10.272, M10.3, M10.319, M10.321, M10.322, M10.329, M10.33, M10.331, M10.332, M10.339, M10.342, M10.349, M10.351, M10.352, M10.359, M10.362, M10.371, M10.379, M10.39, M10.4, M10.412, M10.42, M10.429, M10.431, M10.439, M10.449, M10.451, M10.459, M10.462, M10.469, M10.479, M10.49, M10.9, M11.0, M11.012, M11.022, M11.03, M11.031, M11.041, M11.051, M11.059, M11.061, M11.062, M11.069, M11.07, M11.071, M11.072, M11.079, M11.08, M11.09, M11.11, M11.111, M11.12, M11.121, M11.122, M11.131, M11.132, M11.139, M11.14, M11.141, M11.142, M11.149, M11.159, M11.16, M11.169, M11.172, M11.18, M11.211, M11.22, M11.221, M11.222, M11.232, M11.24, M11.249, M11.25, M11.252, M11.262, M11.269, M11.27, M11.272, M11.279, M11.811, M11.812, M11.822, M11.829, M11.831, M11.832, M11.839, M11.849, M11.85, M11.851, M11.852, M11.86, M11.862, M11.869, M11.87, M11.871, M11.872, M11.879, M11.9, M12, M12.00, M12.011, M12.012, M12.02, M12.022, M12.03, M12.031, M12.032, M12.062, M12.07, M12.1, M12.11, M12.111, M12.112, M12.119, M12.121, M12.14, M12.141, M12.142, M12.149, M12.151, M12.159, M12.161, M12.162, M12.169, M12.17, M12.171, M12.179, M12.18, M12.19, M12.2, M12.219, M12.221, M12.222, M12.242, M12.25, M12.259, M12.26, M12.261, M12.269, M12.27, M12.271, M12.272, M12.28, M12.29, M12.3, M12.31, M12.311, M12.32, M12.321, M12.33, M12.341, M12.342, M12.349, M12.351, M12.352, M12.361, M12.37, M12.371, M12.379, M12.40, M12.41, M12.412, M12.42, M12.422, M12.429, M12.432, M12.439, M12.44, M12.441, M12.442, M12.469, M12.472, M12.49, M12.50, M12.512, M12.519, M12.521, M12.529, M12.53, M12.532, M12.541, M12.559, M12.56, M12.561, M12.562, M12.572, M12.579, M12.58, M12.80, M12.811, M12.82, M12.829, M12.83, M12.831, M12.832, M12.84, M12.841, M12.849, M12.85, M12.852, M12.859, M12.86, M12.861, M12.87, M12.89, M12.9, M13, M13.1, M13.112, M13.119, M13.12, M13.121, M13.122, M13.129, M13.131, M13.132, M13.14, M13.149, M13.15, M13.151, M13.159, M13.16, M13.169, M13.172, M13.81, M13.84, M13.842, M13.849, M13.85, M13.852, M13.859, M13.86, M13.861, M13.871, M13.872, M13.879, M13.88, M13.89, M14.6, M14.61, M14.619, M14.62, M14.621, M14.622, M14.63, M14.631, M14.641, M14.642, M14.65, M14.651, M14.652, M14.66, M14.662, M14.669, M14.672, M14.8, M14.812, M14.821, M14.822, M14.83, M14.832, M14.839, M14.84, M14.842, M14.849, M14.86, M14.861, M14.869, M14.87, M14.871, M14.872, M14.89, M30, M46, R76.1, R76.11, R76.9, R83.8, R84.1, R84.2, R84.3, R84.4, R84.5, R84.7, R84.9, R85, R85.0, R85.1, R85.2, R85.4, R85.5, R85.610, R85.611, R85.613, R85.614, R85.615, R85.616, R85.619, R85.69, R85.7, R86.4, R86.6, R86.8, R87.0, R87.1, R89.4, T86, Z85, Z98.85 |

Table 2. Incidence of Any Observed COVID-19, COVID-19–Related Hospitalizations and All-cause Mortality Temporally Associated with a COVID-19 disease, and Vaccine Effectiveness among Subgroups of Interest

| **Subgroup** | **Events**  **(n)** | | **Median follow-up time**  **(days)** | | **Risk per 1000 patients** | | **Person-years** | **Incidence rate**  **(per 1000 person years)** | | **Events**  **(n)** | | **Median follow-up time**  **(days)** | | **Risk per 1000 patients** | | **Person-years** | **Incidence rate**  **(per 1000 person years)** | | **Hazard Ratio**  **(95% CI)** | **Vaccine Effectiveness**  **(95% CI)** |
| --- | --- | --- | --- | --- | --- | --- | --- | --- | --- | --- | --- | --- | --- | --- | --- | --- | --- | --- | --- | --- |
| **Age < 65** | **Ad26.COV2.S Vaccinated Group**  **(n=443,957)** | | | | | | | | | **PS-Matched Unvaccinated Group**  **(n=1,763,301)** | | | | | | | | | **Observed VE** | |
| Any observed COVID-19 | 34,816 | 254 [200, 365] | | 78.42 | | 312,135 | | | 111.54 | 150,208 | 241 [102, 365] | | 85.19 | | 1,077,467 | | | 139.41 | 0.79 (0.79, 0.80) | 21% (20%, 21%) |
| COVID-19-related hospitalization | 2,812 | 267 [210, 365] | | 6.33 | | 325,003 | | | 8.65 | 18,113 | 261 [115, 365] | | 10.27 | | 1,135,438 | | | 15.95 | 0.53 (0.51, 0.55) | 47% (45%, 49%) |
| All-cause mortality temporally associated with COVID-19 disease | 59 | 268 [210, 365] | | 0.13 | | 326,148 | | | 0.18 | 549 | 265 [118, 365] | | 0.31 | | 1,143,244 | | | 0.48 | 0.37 (0.28, 0.48) | 63% (52%, 72%) |
| **Age >= 65** | **Ad26.COV2.S Vaccinated Group**  **(n=34,205)** | | | | | | | | | **PS-Matched Unvaccinated Group**  **(n=134,170)** | | | | | | | | | **Observed VE** | |
| Any observed COVID-19 | 2,257 | 262 [199, 365] | | 65.98 | | 24,254 | | | 93.06 | 9,164 | 275 [140, 365] | | 68.30 | | 90,024 | | | 101.79 | 0.93 (0.88, 0.97) | 7% (3%, 12%) |
| COVID-19-related hospitalization | 767 | 269 [206, 365] | | 22.42 | | 24,825 | | | 30.90 | 3,454 | 283 [152, 365] | | 25.74 | | 92,341 | | | 37.40 | 0.83 (0.77, 0.90) | 17% (10%, 23%) |
| All-cause mortality temporally associated with COVID-19 disease | 48 | 274 [209, 365] | | 1.40 | | 25,097 | | | 1.91 | 246 | 289 [159, 365] | | 1.83 | | 93,624 | | | 2.63 | 0.74 (0.54, 1.01) | 26% (-1%, 46%) |
| **HIV Positive** | **Ad26.COV2.S Vaccinated Group**  **(n=2,635)** | | | | | | | | | **PS-Matched Unvaccinated Group**  **(n=134,170)** | | | | | | | | | **Observed VE** | |
| Any observed COVID-19 | 215 | 242 [192, 365] | | 81.59 | | 1,785 | | | 120.48 | 777 | 233 [82, 365] | | 75.88 | | 6,075 | | | 127.91 | 0.93 (0.80, 1.09) | 7% (-9%, 20%) |
| COVID-19-related hospitalization | 42 | 251 [201, 365] | | 15.94 | | 1,856 | | | 22.63 | 220 | 252 [90, 365] | | 21.48 | | 6,316 | | | 34.83 | 0.65 (0.47, 0.90) | 35% (10%, 53%) |
| All-cause mortality temporally associated with COVID-19 disease | <5 | 252 [203, 365] | | 0.00 | | 1,875 | | | 0.00 | 7 | 259 [94, 365] | | 0.68 | | 6,411 | | | 1.09 | NR | NR |
| **HIV Negative** | **Ad26.COV2.S Vaccinated Group**  **(n=475,527)** | | | | | | | | | **PS-Matched Unvaccinated Group**  **(n=1,887,243)** | | | | | | | | | **Observed VE** | |
| **HIV Negative** | **Ad26.COV2.S Vaccinated Group**  **(n=475,527)** | | | | | | | | | **PS-Matched Unvaccinated Group**  **(n=1,887,243)** | | | | | | | | | **Observed VE** | |
| Any observed COVID-19 | 36,858 | 255 [200, 365] | | 77.51 | | 334,604 | | | 110.15 | 158,664 | 243 [105, 365] | | 84.07 | | 1,161,198 | | | 136.64 | 0.80 (0.79, 0.81) | 20% (19%, 21%) |
| COVID-19-related hospitalization | 3,537 | 267 [210, 365] | | 7.44 | | 347,972 | | | 10.16 | 21,510 | 263 [118, 365] | | 11.40 | | 1,221,257 | | | 17.61 | 0.57 (0.55, 0.59) | 43% (41%, 45%) |
| All-cause mortality temporally associated with COVID-19 disease | 107 | 268 [210, 365] | | 0.23 | | 349,370 | | | 0.31 | 779 | 268 [121, 365] | | 0.41 | | 1,230,310 | | | 0.63 | 0.48 (0.39, 0.58) | 52% (42%, 61%) |
| **COPD** | **Ad26.COV2.S Vaccinated Group**  **(n=52,392)** | | | | | | | | | **PS-Matched Unvaccinated Group**  **(n=205,142)** | | | | | | | | | **Observed VE** | |
| Any observed COVID-19 | 5,782 | 252 [193, 365] | | 110.36 | | 36,196 | | | 159.74 | 23,837 | 238 [101, 365] | | 116.20 | | 124,645 | | | 191.24 | 0.83 (0.81, 0.86) | 17% (14%, 19%) |
| COVID-19-related hospitalization | 1,074 | 267 [206, 365] | | 20.50 | | 38,085 | | | 28.20 | 5,377 | 265 [117, 365] | | 26.21 | | 132,794 | | | 40.49 | 0.69 (0.65, 0.74) | 31% (26%, 35%) |
| All-cause mortality temporally associated with COVID-19 disease | 44 | 271 [209, 365] | | 0.84 | | 38,517 | | | 1.14 | 251 | 274 [123, 365] | | 1.22 | | 135,012 | | | 1.86 | 0.61 (0.45, 0.85) | 39% (15%, 55%) |
| **No COPD** | **Ad26.COV2.S Vaccinated Group**  **(n=425,770)** | | | | | | | | | **PS-Matched Unvaccinated Group**  **(n=1,692,338)** | | | | | | | | | **Observed VE** | |
| Any observed COVID-19 | 31,291 | 255 [201, 365] | | 73.49 | | 300,192 | | | 104.24 | 135,885 | 244 [105, 365] | | 80.29 | | 1,042,383 | | | 130.36 | 0.80 (0.79, 0.80) | 20% (20%, 21%) |
| COVID-19-related hospitalization | 2,505 | 267 [210, 365] | | 5.88 | | 311,743 | | | 8.04 | 16,349 | 263 [118, 365] | | 9.66 | | 1,094,679 | | | 14.93 | 0.53 (0.51, 0.55) | 47% (45%, 49%) |
| All-cause mortality temporally associated with COVID-19 disease | 63 | 268 [210, 365] | | 0.15 | | 312,728 | | | 0.20 | 545 | 267 [120, 365] | | 0.32 | | 1,101,593 | | | 0.49 | 0.40 (0.31, 0.52) | 60% (48%, 69%) |
| **Type 1 Diabetes** | **Ad26.COV2.S Vaccinated Group**  **(n=4,575)** | | | | | | | | | **PS-Matched Unvaccinated Group**  **(n=17,740)** | | | | | | | | | **Observed VE** | |
| Any observed COVID-19 | 517 | 251 [193, 365] | | 113.01 | | 3,147 | | | 164.29 | 2,126 | 234 [102, 365] | | 119.84 | | 10,727 | | | 198.19 | 0.83 (0.75, 0.91) | 17% (9%, 25%) |
| COVID-19-related hospitalization | 155 | 264 [204, 365] | | 33.88 | | 3,292 | | | 47.08 | 745 | 258 [115, 365] | | 42.00 | | 11,334 | | | 65.73 | 0.72 (0.60, 0.85) | 28% (15%, 40%) |
| All-cause mortality temporally associated with COVID-19 disease | 8 | 269 [209, 365] | | 1.75 | | 3,351 | | | 2.39 | 44 | 271 [123, 365] | | 2.48 | | 11,647 | | | 3.78 | 0.64 (0.30, 1.35) | 36% (-35%, 70%) |
| **No Type 1 Diabetes** | **Ad26.COV2.S Vaccinated Group**  **(n=473,585)** | | | | | | | | | **PS-Matched Unvaccinated Group**  **(n=1,879,697)** | | | | | | | | | **Observed VE** | |
| Any observed COVID-19 | 36,556 | 255 [200, 365] | | 77.19 | | 333,240 | | | 109.70 | 157,423 | 243 [105, 365] | | 83.75 | | 1,156,440 | | | 136.13 | 0.80 (0.79, 0.81) | 20% (19%, 21%) |
| COVID-19-related hospitalization | 3,424 | 267 [209, 365] | | 7.23 | | 346,535 | | | 9.88 | 20,922 | 263 [118, 365] | | 11.13 | | 1,216,204 | | | 17.20 | 0.57 (0.55, 0.59) | 43% (41%, 45%) |
| All-cause mortality temporally associated with COVID-19 disease | 99 | 268 [210, 365] | | 0.21 | | 347,892 | | | 0.28 | 740 | 268 [120, 365] | | 0.39 | | 1,225,013 | | | 0.60 | 0.46 (0.38, 0.57) | 54% (43%, 62%) |
| **Type 2 Diabetes** | **Ad26.COV2.S Vaccinated Group**  **(n=59,192)** | | | | | | | | | **PS-Matched Unvaccinated Group**  **(n=231,969)** | | | | | | | | | **Observed VE** | |
| Any observed COVID-19 | 5,977 | 253 [197, 365] | | 100.98 | | 41,036 | | | 145.65 | 23,718 | 236 [98, 365] | | 102.25 | | 139,800 | | | 169.66 | 0.86 (0.84, 0.88) | 14% (12%, 16%) |
| COVID-19-related hospitalization | 1,330 | 264 [208, 365] | | 22.47 | | 42,900 | | | 31.00 | 6,505 | 257 [109, 365] | | 28.04 | | 147,266 | | | 44.17 | 0.70 (0.66, 0.74) | 30% (26%, 34%) |
| All-cause mortality temporally associated with COVID-19 disease | 50 | 268 [211, 365] | | 0.84 | | 43,423 | | | 1.15 | 316 | 266 [115, 365] | | 1.36 | | 149,909 | | | 2.11 | 0.55 (0.40, 0.74) | 45% (26%, 60%) |
| **No Type 2 Diabetes** | **Ad26.COV2.S Vaccinated Group**  **(n=418,970)** | | | | | | | | | **PS-Matched Unvaccinated Group**  **(n=1,665,351)** | | | | | | | | | **Observed VE** | |
| Any observed COVID-19 | 31,096 | 255 [200, 365] | | 74.22 | | 295,352 | | | 105.28 | 135,853 | 244 [106, 365] | | 81.58 | | 1,027,196 | | | 132.26 | 0.79 (0.78, 0.80) | 21% (20%, 22%) |
| COVID-19-related hospitalization | 2,249 | 267 [210, 365] | | 5.37 | | 306,928 | | | 7.33 | 15,151 | 264 [119, 365] | | 9.10 | | 1,080,128 | | | 14.03 | 0.51 (0.49, 0.54) | 49% (46%, 51%) |
| All-cause mortality temporally associated with COVID-19 disease | 57 | 268 [210, 365] | | 0.14 | | 307,821 | | | 0.19 | 464 | 268 [121, 365] | | 0.28 | | 1,086,621 | | | 0.43 | 0.43 (0.32, 0.56) | 57% (44%, 68%) |
| **Chronic Neurological Disorders** | **Ad26.COV2.S Vaccinated Group**  **(n=16,376)** | | | | | | | | | **PS-Matched Unvaccinated Group**  **(n=63,895)** | | | | | | | | | **Observed VE** | |
| Any observed COVID-19 | 1,634 | 255 [190, 365] | | 99.78 | | 11,338 | | | 144.12 | 6,975 | 248 [102, 365] | | 109.16 | | 39,423 | | | 176.93 | 0.82 (0.77, 0.86) | 18% (14%, 23%) |
| COVID-19-related hospitalization | 521 | 268 [200, 365] | | 31.81 | | 11,794 | | | 44.18 | 2,544 | 269 [113, 365] | | 39.82 | | 41,403 | | | 61.45 | 0.72 (0.66, 0.79) | 28% (21%, 34%) |
| All-cause mortality temporally associated with COVID-19 disease | 15 | 275 [205, 365] | | 0.92 | | 12,016 | | | 1.25 | 105 | 282 [123, 365] | | 1.64 | | 42,480 | | | 2.47 | 0.49 (0.28, 0.84) | 51% (16%, 72%) |
| **No Chronic Neurological Disorders** | **Ad26.COV2.S Vaccinated Group**  **(n=461,786)** | | | | | | | | | **PS-Matched Unvaccinated Group**  **(n=1,833,622)** | | | | | | | | | **Observed VE** | |
| Any observed COVID-19 | 35,439 | 255 [200, 365] | | 76.74 | | 325,051 | | | 109.03 | 152,578 | 243 [105, 365] | | 83.21 | | 1,128,020 | | | 135.26 | 0.80 (0.79, 0.81) | 20% (19%, 21%) |
| COVID-19-related hospitalization | 3,058 | 267 [210, 365] | | 6.62 | | 338,034 | | | 9.05 | 19,029 | 263 [118, 365] | | 10.38 | | 1,186,462 | | | 16.04 | 0.56 (0.54, 0.58) | 44% (42%, 46%) |
| All-cause mortality temporally associated with COVID-19 disease | 92 | 268 [210, 365] | | 0.20 | | 339,228 | | | 0.27 | 661 | 267 [120, 365] | | 0.36 | | 1,194,468 | | | 0.55 | 0.48 (0.39, 0.60) | 52% (40%, 61%) |
| **Cardiovascular Disease** | **Ad26.COV2.S Vaccinated Group**  **(n=158,143)** | | | | | | | | | **PS-Matched Unvaccinated Group**  **(n=622,091)** | | | | | | | | | **Observed VE** | |
| Any observed COVID-19 | 14,557 | 253 [198, 365] | | 92.05 | | 110,398 | | | 131.86 | 60,306 | 240 [103, 365] | | 96.94 | | 379,467 | | | 158.92 | 0.83 (0.81, 0.84) | 17% (16%, 19%) |
| COVID-19-related hospitalization | 2,442 | 265 [209, 365] | | 15.44 | | 115,261 | | | 21.19 | 12,962 | 261 [116, 365] | | 20.84 | | 400,110 | | | 32.40 | 0.65 (0.62, 0.68) | 35% (32%, 38%) |
| All-cause mortality temporally associated with COVID-19 disease | 92 | 268 [211, 365] | | 0.58 | | 116,225 | | | 0.79 | 592 | 268 [120, 365] | | 0.95 | | 405,455 | | | 1.46 | 0.54 (0.43, 0.67) | 46% (33%, 57%) |
| **No Cardiovascular Disease** | **Ad26.COV2.S Vaccinated Group**  **(n=320,019)** | | | | | | | | | **PS-Matched Unvaccinated Group**  **(n=1,275,414)** | | | | | | | | | **Observed VE** | |
| Any observed COVID-19 | 22,516 | 256 [201, 365] | | 70.36 | | 225,990 | | | 99.63 | 99,148 | 246 [106, 365] | | 77.74 | | 787,767 | | | 125.86 | 0.79 (0.77, 0.80) | 21% (20%, 23%) |
| COVID-19-related hospitalization | 1,137 | 267 [210, 365] | | 3.55 | | 234,567 | | | 4.85 | 8,661 | 264 [119, 365] | | 6.79 | | 827,477 | | | 10.47 | 0.46 (0.43, 0.49) | 54% (51%, 57%) |
| All-cause mortality temporally associated with COVID-19 disease | 15 | 268 [210, 365] | | 0.05 | | 235,020 | | | 0.06 | 182 | 267 [120, 365] | | 0.14 | | 831,234 | | | 0.22 | 0.29 (0.17, 0.49) | 71% (51%, 83%) |
| **Cerebrovascular Disease** | **Ad26.COV2.S Vaccinated Group**  **(n=6,243)** | | | | | | | | | **PS-Matched Unvaccinated Group**  **(n=24,426)** | | | | | | | | | **Observed VE** | |
| Any observed COVID-19 | 644 | 252 [189, 365] | | 103.16 | | 4,278 | | | 150.54 | 2,790 | 239 [97, 365] | | 114.22 | | 14,743 | | | 189.24 | 0.80 (0.74, 0.88) | 20% (12%, 26%) |
| COVID-19-related hospitalization | 197 | 264 [200, 365] | | 31.56 | | 4,462 | | | 44.15 | 1,119 | 259 [106, 365] | | 45.81 | | 15,499 | | | 72.20 | 0.62 (0.53, 0.72) | 38% (28%, 47%) |
| All-cause mortality temporally associated with COVID-19 disease | 10 | 271 [204, 365] | | 1.60 | | 4,546 | | | 2.20 | 58 | 274 [116, 365] | | 2.37 | | 15,946 | | | 3.64 | 0.60 (0.31, 1.17) | 40% (-17%, 69%) |
| **No Cerebrovascular Disease** | **Ad26.COV2.S Vaccinated Group**  **(n=471,919)** | | | | | | | | | **PS-Matched Unvaccinated Group**  **(n=1,873,159)** | | | | | | | | | **Observed VE** | |
| Any observed COVID-19 | 36,429 | 255 [200, 365] | | 77.19 | | 332,110 | | | 109.69 | 156,731 | 243 [105, 365] | | 83.67 | | 1,152,414 | | | 136.00 | 0.80 (0.79, 0.81) | 20% (19%, 21%) |
| COVID-19-related hospitalization | 3,382 | 267 [210, 365] | | 7.17 | | 345,366 | | | 9.79 | 20,576 | 263 [118, 365] | | 10.98 | | 1,212,023 | | | 16.98 | 0.57 (0.55, 0.59) | 43% (41%, 45%) |
| All-cause mortality temporally associated with a COVID-19 disease | 97 | 268 [210, 365] | | 0.21 | | 346,698 | | | 0.28 | 721 | 267 [120, 365] | | 0.38 | | 1,220,711 | | | 0.59 | 0.47 (0.38, 0.58) | 53% (42%, 62%) |

## Figure 1. Study design diagram


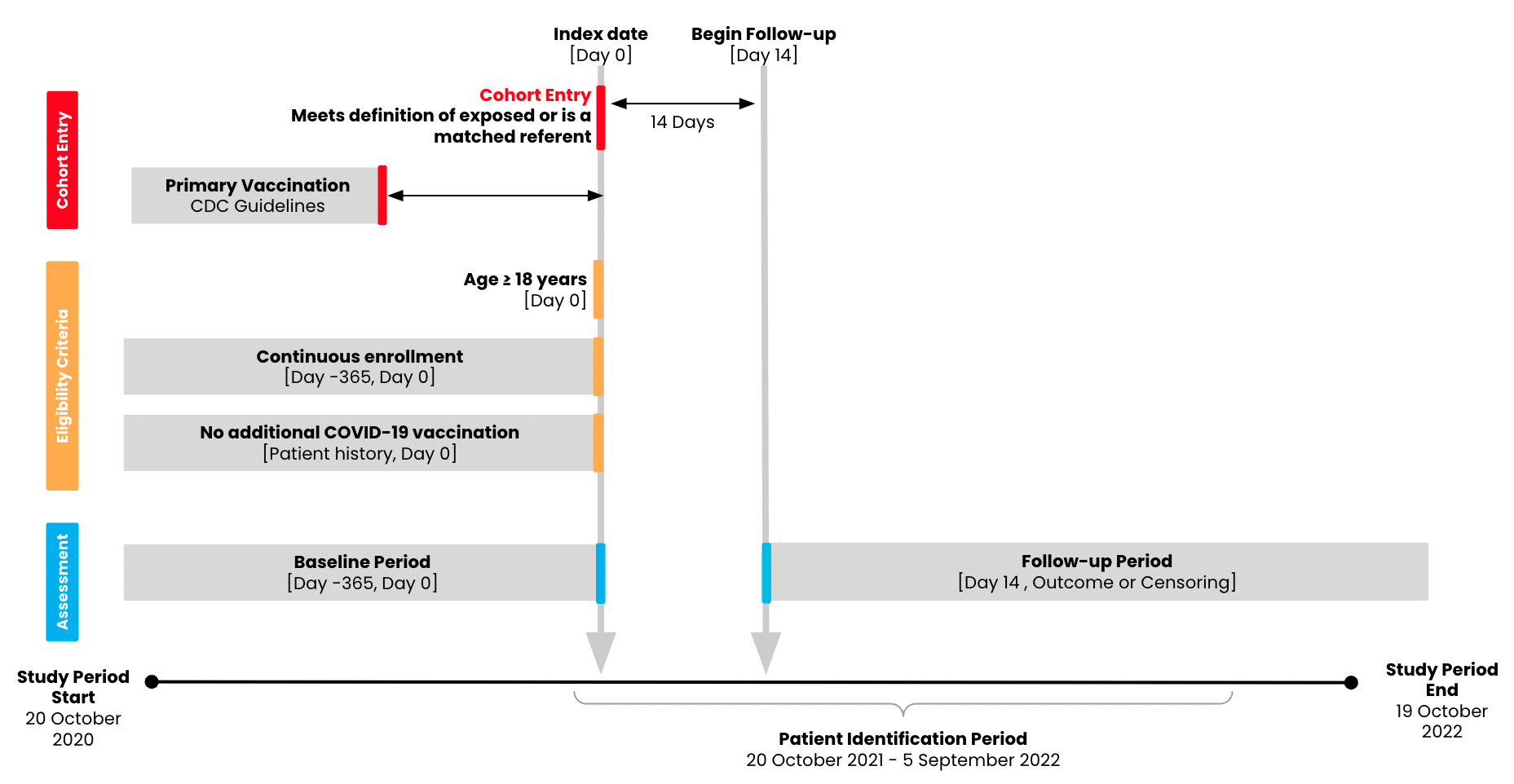

Supplement: Supplementary file 1 [file Data_Sheet_1.docx]
